# Tissue Phenomics for prognostic biomarker discovery in low- and intermediate-risk prostate cancer

**DOI:** 10.1038/s41598-018-22564-7

**Published:** 2018-03-13

**Authors:** Nathalie Harder, Maria Athelogou, Harald Hessel, Nicolas Brieu, Mehmet Yigitsoy, Johannes Zimmermann, Martin Baatz, Alexander Buchner, Christian G. Stief, Thomas Kirchner, Gerd Binnig, Günter Schmidt, Ralf Huss

**Affiliations:** 1 Definiens AG, Munich, Germany; 2 Institute for Pathology, Ludwig-Maximilians-University, Munich, Germany; 3 Department of Urology, Ludwig-Maximilians-University, Munich, Germany; 4 Present Address: Carl Zeiss Meditec AG, Munich, Germany

## Abstract

Tissue Phenomics is the discipline of mining tissue images to identify patterns that are related to clinical outcome providing potential prognostic and predictive value. This involves the discovery process from assay development, image analysis, and data mining to the final interpretation and validation of the findings. Importantly, this process is not linear but allows backward steps and optimization loops over multiple sub-processes. We provide a detailed description of the Tissue Phenomics methodology while exemplifying each step on the application of prostate cancer recurrence prediction. In particular, we automatically identified tissue-based biomarkers having significant prognostic value for low- and intermediate-risk prostate cancer patients (Gleason scores 6–7b) after radical prostatectomy. We found that promising phenes were related to CD8(+) and CD68(+) cells in the microenvironment of cancerous glands in combination with the local micro-vascularization. Recurrence prediction based on the selected phenes yielded accuracies up to 83% thereby clearly outperforming prediction based on the Gleason score. Moreover, we compared different machine learning algorithms to combine the most relevant phenes resulting in increased accuracies of 88% for tumor progression prediction. These findings will be of potential use for future prognostic tests for prostate cancer patients and provide a proof-of-principle of the Tissue Phenomics approach.

## Introduction

Tissue Phenomics is the systematic discovery of quantitative descriptors for functional, morphological and spatial patterns in tissue which correlate with disease progression or drug response. It complements genomics which links genetic information and disease^1^ by adding information on protein expression levels, cellular interactions and architectures. Using the latest whole slide imaging hardware, advanced multiparametric image analysis, and big data knowledge discovery, Tissue Phenomics may supersede the manual discovery of novel scoring algorithms in histopathology. Since the extracted quantitative descriptors can be stored in large databases and cross-linked with other biomedical knowledge, Tissue Phenomics also stimulates clinical research, improves clinical drug development and the treatment of patients. Novel image-based biomarkers, referred to as *tissue phenes*, leverage pathologists’ scientific and clinical insight into the information contained in tissue. Therefore, it has potential to impact decisively on medicine in general and on oncology in particular.

Examples of novel tissue- and image-based biomarkers have been described in previous work, e.g. refs^2–4^. While Beck *et al*.^2^ followed a hypothesis-free approach, for Galon *et al*.^3^ and Caie *et al*.^4^ knowledge about a potential correlation of certain tissue structures and their properties with clinical outcome already existed. However, in all three cases quantification and statistical evaluation led to surprising results that could not have been achieved by manual approaches. In Galon *et al*.^3^ it was known that the immune system responds to tumor tissue, but it was not anticipated that the immune response could be described by a relative simple mathematical formalism with a high prognostic value. For Caie *et al*.^4^ budding tumor cells in the invasive margin of colon cancers were known to have a meaning for the prognosis of patients (e.g. refs^5,6^), but the high prognostic value of big structures of buds was discovered only by using Tissue Phenomics. In the work of Beck *et al*.^2^ structural features significantly associated with survival in breast cancer were discovered in the stroma close to the tumor by quantification and data mining in an unbiased data-driven Tissue Phenomics approach.

Other related approaches identified prognostic image-based biomarkers based on, e.g., nuclear morphometric features in male breast cancer^7^, spatial arrangement patterns of stromal cells in breast cancer^8^ and of tumor cells in oropharyngeal squamous cell carcinomas^9^, or glandular orientation patterns in prostate cancer^10^. However, these approaches were based on relatively clearly sketched initial hypotheses, therefore operating on specific and targeted feature sets. This naturally limits the ability of discovering novel and unexpected image-based biomarkers for creating new biological insights which is a main objective of Tissue Phenomics. For a more general review of approaches for predictive modeling in histopathological images see, for example^11^.

In this study, we used the Tissue Phenomics approach to identify novel prognostic biomarkers for pT2-staged prostate cancer patients who underwent radical prostatectomy with negative resection border. Subsequent to surgery those biomarkers support the selection between more aggressive treatment options such as radiation therapy and more conservative options such as watchful waiting. The clinical endpoint used in the discovery process was the prostate-specific antigen (PSA) recurrence. Since the Gleason grading is of limited prognostic value for low and intermediate-risk patients (Gleason score <= 7b)^12^ we included in our study immune contexture-related information such as markers for CD8(+) cytotoxic T-cells as well as CD68(+) M1 and CD163(+) M2 macrophages. Inspired by the Immunoscore for colon cancer patients^3^ we investigated the spatial relationship of the various immune populations within cancer, stromal and benign regions. The comprehensive screening for prognostic signatures based on multi-parametric sets of contextual cell density measurements sets this study apart from recent investigations on single aspects of certain cell populations (e.g. ref.^13^).

## Material and Methods

### The Tissue Phenomics approach

**Figure 1:**
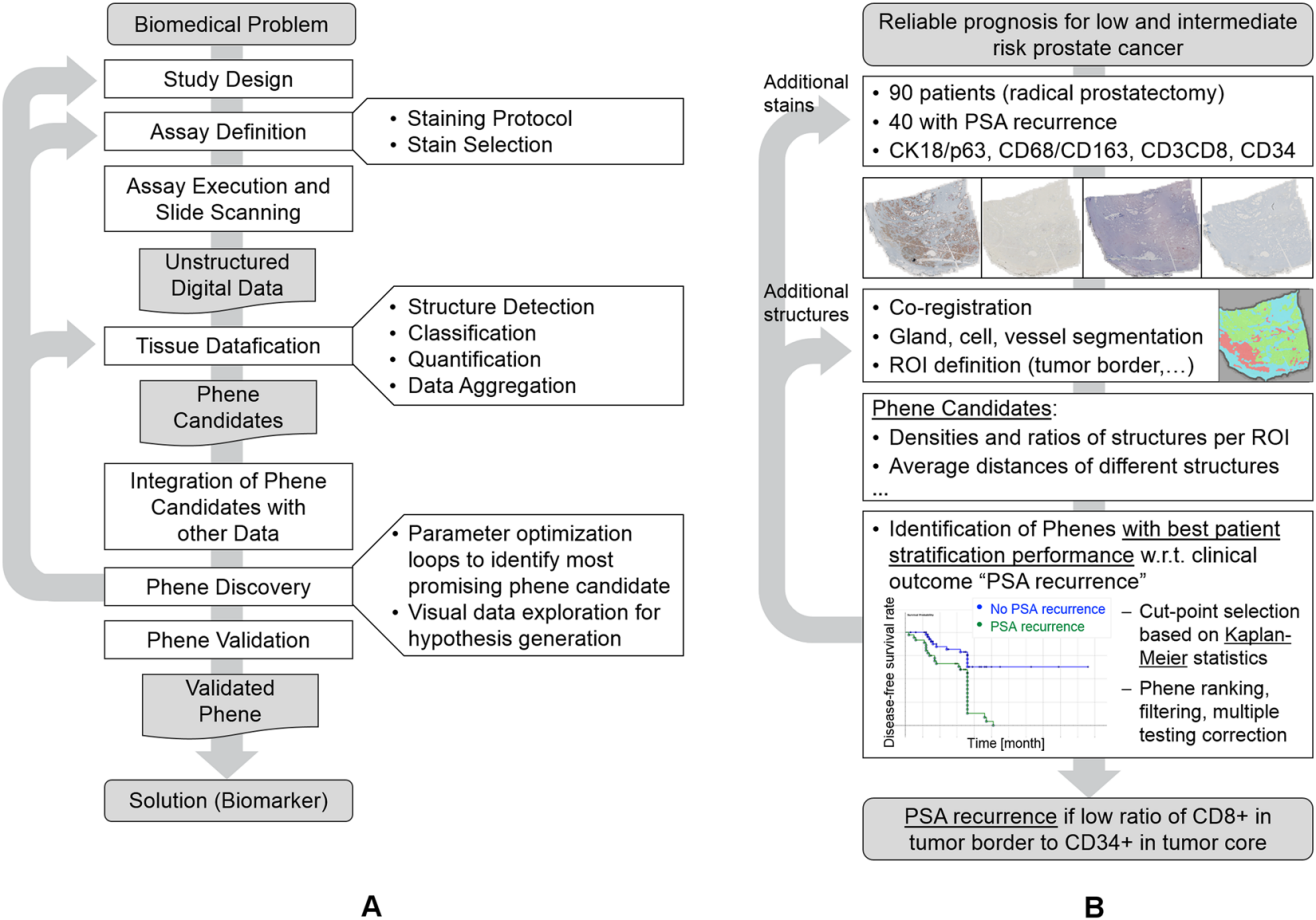
The Tissue Phenomics workflow. (**A**) General workflow: Tissue Phenomics is a Big Data approach for quantitative histopathology, spanning from assay development to phene validation based on automated multi-parametric optimization. (**B**) Example application: Identification of phenes for improved prognosis for low- and intermediate-risk prostate cancer patients after radical prostatectomy. Note that in this example the phene validation step is not yet fully covered. (For algorithm descriptions see supplemental material, flow charts).

The core of the Tissue Phenomics methodology is an iterative optimization workflow comprising experimental and lab-related steps such as study design, assay development, tissue acquisition, staining and digitization, as well as advanced image and data analysis for phene discovery and optimization. A final validation step ensures the bio-statistical and biomedical validity of the biomarkers represented by the discovered phenes (see Fig. 1A). In this paper, we use the example application on prostate cancer recurrence prediction to describe the Tissue Phenomics workflow (see Fig. 1B). For every step we first provide a general description followed by the concrete application example.

### Study design and execution

#### Study design

In the study design process, the general conditions and parameters of the study are defined, such as number and type of cases, as well as the type and selection criteria of tissue samples. As certain parameters or conditions might turn out to be not optimally chosen throughout the progression and evaluation of the study, all parameters are eligible for later optimization regarding the overall solution quality through multiple iterations of a feedback loop.

The prostate cancer study included 90 low- and intermediate-risk prostate cancer patients (Gleason-Score ≤ 7b, age <= 75 years, staging pT2, resection border R0)^14^. All patients underwent radical prostatectomy and a follow-up of the patients within a period of 2–118 months was available where 40 patients developed prostate-specific antigen (PSA) biochemical recurrence, indicating tumor progression (disease-free survival mean = 25.8 months, median = 31.5 months), see Table 1. Standard clinical data such as Gleason score, cancer stage, pre-operative PSA value, and age did not allow accurate prediction of disease progression as shown by survival analysis and Cox regression (see supplemental Figs S1, S2, and Table S1).

**Table 1:** Properties of the prostate cancer patient cohort.

Total number of patients	90			
Patients with PSA recurrence	40			
**Gleason score** Number of patients	**6** 49	**7a** 31		**7b** 10
**Stage pT** Number of patients	**2a** 13	**2b** 1		**2c** 76
**Patient age** (>=75y) Number of patients	**40–55** y 12	**56–65** y 39		**66–75** y 39
Disease-free survival time (for non-censored patients)	**mean** 25.8 months		**median** 31.5 months	
Overall follow-up period	2–118 months			

The goal of the study was to identify phene candidates providing a reliable prognosis of disease progression based on serially cut and immunohistochemically stained tissue sections using biomarkers for epithelial, endothelial, basal, and immune cells. For the ethical approval of the study see end of section Material and Methods below.

#### Assay definition

A *tissue phene* can be defined as a quantified characteristic of a structure in formalin-fixed paraffin-embedded (FFPE) or fresh frozen tissue sections. In a broader definition, also radiological images of tissue can be included, however, in this work we focus on microscopic investigations. The right choice of staining methods is critical for the success of phene discovery in the context of a specific application. Relevant criteria are, for example, the selection of proteins and genes, and of corresponding probes and stains, robustness of the assay, and the deployment model in a clinical environment. In a typical setup for phene discovery, the assay initially consists of a defined set of stains which, however, will potentially not all be relevant for answering the biomedical question. On the other hand, throughout the data mining process new hypotheses might be generated, suggesting to add other proteins to the assay. Such adaptations can be addressed in multiple optimization iterations of the workflow in Fig. 1. Another consideration is the choice between serial sections in brightfield immunohistochemistry or immunofluorescence. Highly multiplexed immunofluorescence may be preferable due to its rich information content and perfect spatial alignment, however, more economical brightfield methods may as well provide sufficient spatial information and accuracy given recent advances of automatic co-registration approaches (see, e.g. refs^15–17^).

For the prostate cancer study, a set of three dual stains (CK18/p63, CD68/CD163, CD3/CD8) and one single stain (CD34) emerged from the Tissue Phenomics optimization by showing relevant prognostic potential regarding cancer recurrence. Other initially involved stains (e.g., CD45RO, CD20) were removed from the phene discovery workflow, as they showed limited relevance for prostate cancer progression. Also, single stains (e.g., CD3, CD8) were replaced by dual stains (CD3/CD8). The stains were selected to provide spatial and structural information, as well as functional information with respect to the immune status of the tumor. Structural information for the annotation of cancerous and non-cancerous tissue was gained from CK18/p63. CK18 visualizes epithelial tissue providing a clear outline of the prostate glandular system, while p63 gives information on whether the basal membrane enclosing a gland is intact or non-intact. The basal epithelial cells of an intact basal membrane express p63, while cells of a corrupted basal membrane lack this protein. Thus, p63 was used as a direct marker for separating cancer tissue from non-cancer tissue in prostate. All other stains were selected to provide information on the local immune status of the investigated tissue, in particular, to monitor interactions of important players, such as CD68/CD163 corresponding to M1 and M2 macrophages, CD3/CD8 expressed by all T-cells (CD3) and cytotoxic T-cells (CD8), as well as CD34 visualizing the micro vasculature.

#### Assay execution and slide scanning

According to the study design and the assay definition tissue is collected, fixated, processed, and stained. Highly standardized procedures are recommended for these steps to generate relevant and comparable data and facilitate information extraction in the datafication step^18^. For slide scanning high-throughput whole-slide scanners are used allowing a high degree of automation.

In the prostate cancer study consecutive formalin-fixed, paraffin-embedded tissue (FFPET) sections were derived from the resected prostate tissue. Immunohistochemistry was performed according to standard protocols using the Ventana Benchmark technology to achieve reproducible and reliable immunohistochemistry test results by standardization of every step in preanalytic, analytic, and postanalytic phases^19^. Whole FFPET sections cut at 3 µm were stained with a Ventana Benchmark XT autostainer (Ventana Medical Systems). Details of the antibodies and methods employed are given in supplemental Table S2. Slides were counterstained with haematoxylin (Ventana). System and isotype controls were included. All sections were imaged using a ZEISS Axio Scan.Z1 tissue scanner (Carl *Zeiss*, Jena, Germany) using a 20x objective (resolution 0.22 μm/pixel). At minimum four digital RGB whole-slide images per patients with around 100 k × 100 k pixels were acquired (i.e. 1–2 GB per image after compression, see supplemental Fig. S3).

### Image analysis for tissue datafication

#### Datafication workflow

The goal of tissue datafication is to extract information from digital images providing a set of phene candidates. The typical datafication workflow comprises (I) image normalization, (II) structure-of-interest detection, (III) co-registration of consecutive tissue sections, (IV) statistical region-based data aggregation and quantification. Note that the order of steps (II) and (III) is of relevance only in specific cases, e.g., if the co-registration relies on the detected structures-of-interest or, vice-versa, if the structure-of-interest detection in one tissue section makes use of structures from another tissue section.

**Figure 2:**
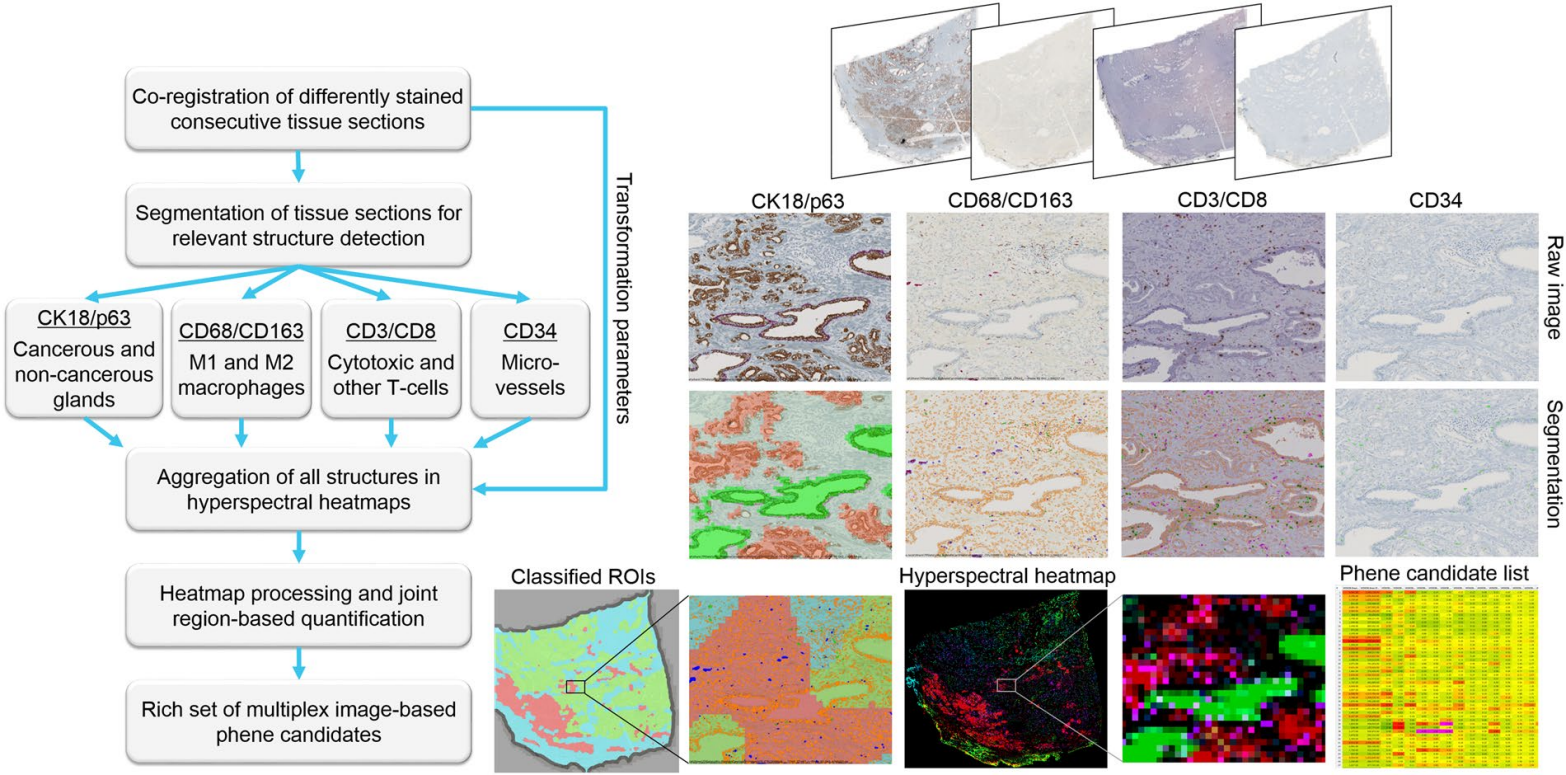
Datafication workflow for the prostate cancer project.

The datafication workflow for the prostate cancer study is shown in Fig. 2. First, image co-registration was performed on the digitized tissue slides, followed by the detection of structures predefined from domain knowledge as being relevant (e.g., cell nuclei, glands, vessel segments). Next, the extracted information was combined in hyper-spectral heatmaps using the geometric transformation parameters obtained by co-registration, and finally, the heatmaps were further processed, for example, to identify cancerous vs. non-cancerous tissue and quantified considering the identified regions-of-interest (ROIs). All steps are described in the following sections.

**Figure 3:**
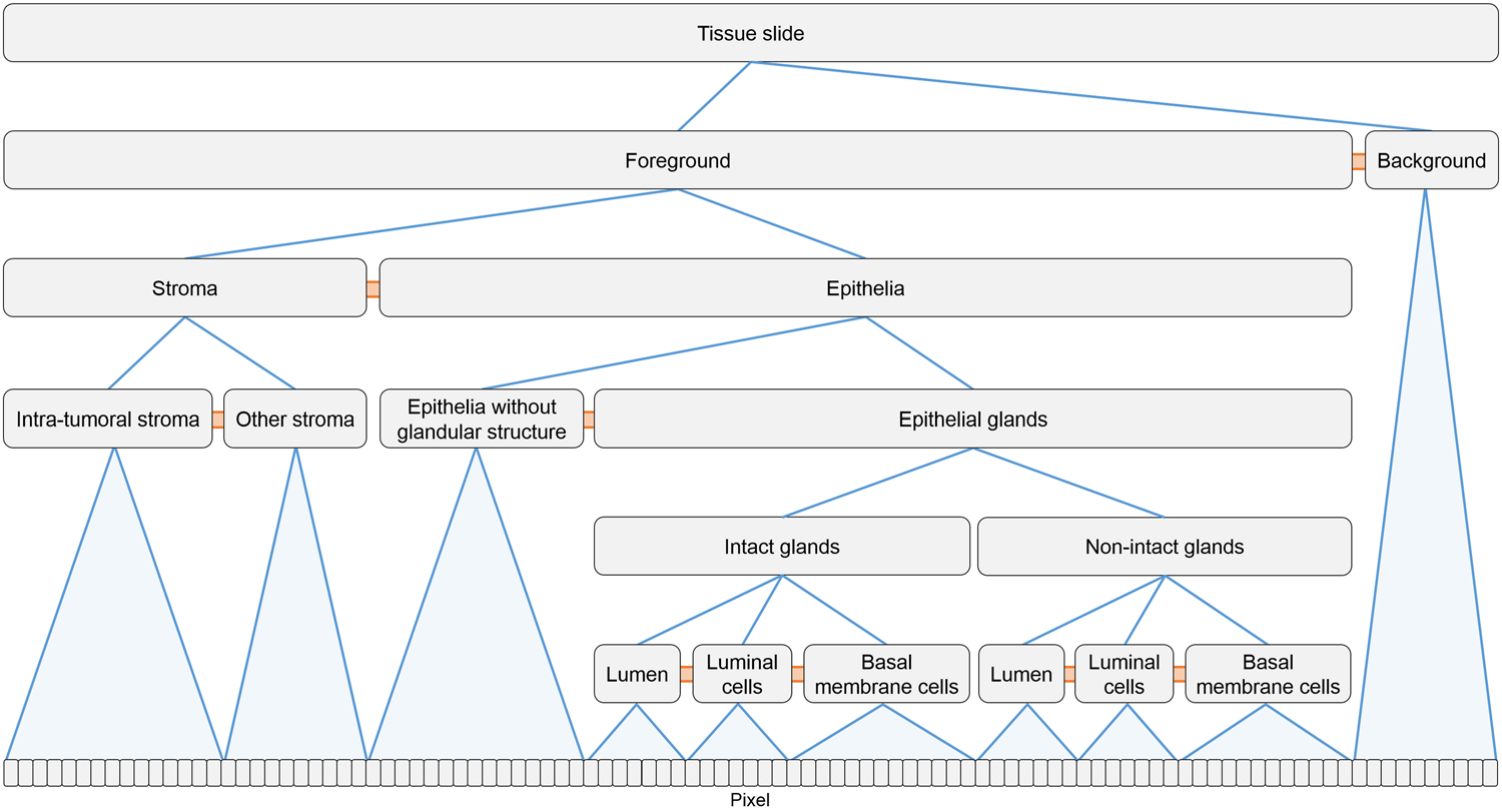
Schematic representation of the network of relevant objects detected in CK18/p63 tissue slides with hierarchical “consists-of” relations (in blue), and non-hierarchical “is-neighbor-of” relations (in orange).
  In our prostate cancer example the datafication step involved detection of marker-positive cells in sections stained for CD3/CD8 and CD68/CD163, detection and classification of glands in CK18/p63, as well as detection of vessel segments in CD34 (Fig. 2). All object detection approaches were developed in the CNL programming framework.
  Gland and vessel segment detection: In images stained for CK18 glands were characterized by a brown-stained cell layer surrounding glandular lumina (see Fig. 4A). For gland segmentation, first luminal epithelial cells were detected based on a generic brown stain detection. Next, luminal region candidates were identified based on a fixed brightness threshold and morphological operations, and filtered with respect to the existence of neighboring epithelial cells taking advantage of the image object network. Thus, only luminal regions surrounded by epithelial cells were identified as glandular lumen. The resulting gland candidates were refined and split into neighboring glands using morphological operations and network relations. To classify glands into healthy glands (with intact basal membrane) and cancerous glands (with non-intact basal membrane), a segmentation of the red-stained p63(+) cell nuclei was performed and the CNL network’s neighborhood relations of glands and p63(+) cells were used for gland classification (Fig. 4). For algorithm details see supplemental material, flow chart 1.

1. Image normalization
  In histopathological image analysis, normalization often is required due to the relatively large variability of the data. Stain variations are caused, for example, by use of different scanners or staining protocols, varying stain intensities or tissue slice thickness. Consequently, a broad variety of normalization approaches have been developed in this field. Basic general purpose techniques are, for example, based on the image histogram and perform histogram normalization (min-max normalization), histogram equalization, or mapping of histograms (by transferring gray value means and standard deviations)^20^ within each color channel. However, such techniques are based on the assumption that the content of all images to be normalized is relatively similar, and thus, histograms are generally comparable. More advanced approaches are based on color deconvolution^21^, and use, for example, singular value decomposition^22^, independent component analysis^23^ or blind color decomposition^24^. For immunohistochemical stains, in particular, approaches based on non-negative matrix factorization (NMF)^25^ have been shown to be successful, such as sparse NMF^26^ or structure-preserving color normalization^27^. For a comparison of stain normalization approaches see, e.g. refs^28,29^. In the prostate cancer example a specific stain normalization step was not required since our methods for segmentation and image registration inherently account for stain variations and the data variability was generally low. In related work we used, e.g., histogram equalization^30^.
2. Structure-of-interest detection and classification
  Hierarchical structure detection using CNL: A key challenge of datafication is the automated detection of relevant structures in the images such as cell nuclei, cells, glands or other structural units, or different tissue architectures. The heterogeneity and variability of tissue types and structures, the section level and thickness, as well as staining issues are major challenges. Therefore, correct identification of structures typically requires incorporating domain knowledge on the structures-of-interest, for example, using model-based approaches with implicit knowledge representation or by explicitly formulating selection criteria (see, e.g. refs^31,32^). An effective approach to formulate object detection algorithms and generate object hierarchies while incorporating domain knowledge is the Cognition Network Language (CNL), a visual scripting language developed to implement the Cognition Network Technology (CNT^33^).
  In CNL, object detection is formulated as an iterative optimization process, grouping pixels to objects and objects to object groups, and refining groups by re-segmentation, group merging or splitting. Alternation of local segmentation operations with local classification steps allows successive extraction of information while optimizing the state of the network of objects until all structures-of-interest are correctly represented. CNL supports a hierarchical organization of structures using “consists-of” relations (e.g., tissue, tumor, viable tumor, tumor cells, cell membrane, see Fig. 3). The network provides context information for the contained structures which is used to support difficult or ambiguous decisions. Explicit expert knowledge characterizing structures-of-interest is included, e.g., using fuzzy logic memberships functions^34–36^. For more complex cases where the relevant structure descriptors cannot be explicitly formulated, models with implicit knowledge representation can be incorporated using machine learning methods for classification such as Random Forests or Deep Learning algorithms.

**Figure 4:**
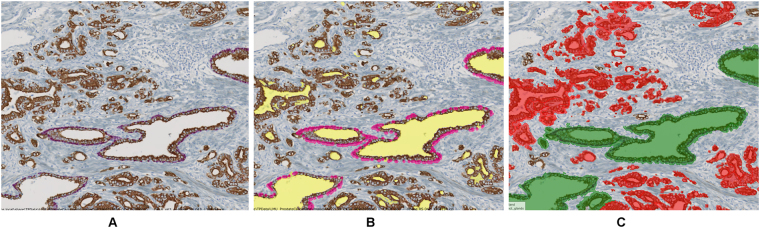
Illustration of the algorithm for gland segmentation. (**A**) CK18/p63 raw image, (**B**) segmentation of lumina (yellow) and p63(+) cells (magenta), (**C**) whole gland segmentation and classification into healthy (green) and cancerous (red) glands according to the existence of neighboring p63(+) cells.
  Vessel segment detection was performed using a similar brown stain detection method as used for the luminal epithelial cells. The detected segments were post-processed using morphological operations to derive meaningful vessel objects.
  Cell and cell nucleus detection: The approach applied for cell detection^37^ is based on the assumption that cell objects underlie a certain morphological consistency and a few well-differentiated representatives of the objects are present in the images. First, well-differentiated objects are automatically extracted based on morphological criteria (size, shape, color constraints), selecting clear and unambiguous cases only from a subset of representative image sections. Second, the detected objects are used to learn the visual appearance of (I) the current image set (with respect to staining, cutting, and digitization), and (II) the characteristic visual context of the objects-of-interest which also applies for low-differentiated objects (e.g., clustered nuclei). The visual context model is based on Random Forests using Long-Range features^38^. Finally, the model is applied to the whole-slide images to predict cell candidates, which are filtered and refined resulting in a robust segmentation. Note that no manual annotation is required with this approach. For algorithm details see supplemental material, flow chart 2. In the prostate cancer study we used the cell detection to identify CD68(+) and CD163(+) cells, corresponding to tumor-associated macrophages (M1 and M2, respectively), as well as CD3(+) and CD8(+)-expressing T-cells, and all marker-negative cell nuclei (see Sect. Results below).
3. Image co-registration
  To jointly study different structural or functional entities (e.g., different cell types) serial sections are stained with a panel of multiple biomarkers. Co-registration of serial sections (virtual multiplexing) enables quantification of spatial relations between objects detected in different sections. However, for immunohistochemically stained sections co-registration is particularly challenging since the characteristics of different stains are typically highly dissimilar for different stains and purely intensity-based methods are not directly applicable (multi-modal registration problem). To deal with multi-modality, macroscopic landmarks with semantic meaning, such as previously detected vessels or glands which are similar in possibly many sections can be used for alignment. Other challenges are artifacts such as tissue folds, ruptured tissue or lacking tissue parts, mirrored tissue sections resulting from flipping during the slide preparation, or non-consecutive sections leading to a lack of corresponding structures between sections (for an example see Fig. 5, panel D vs. panels A-C).

**Figure 5:**
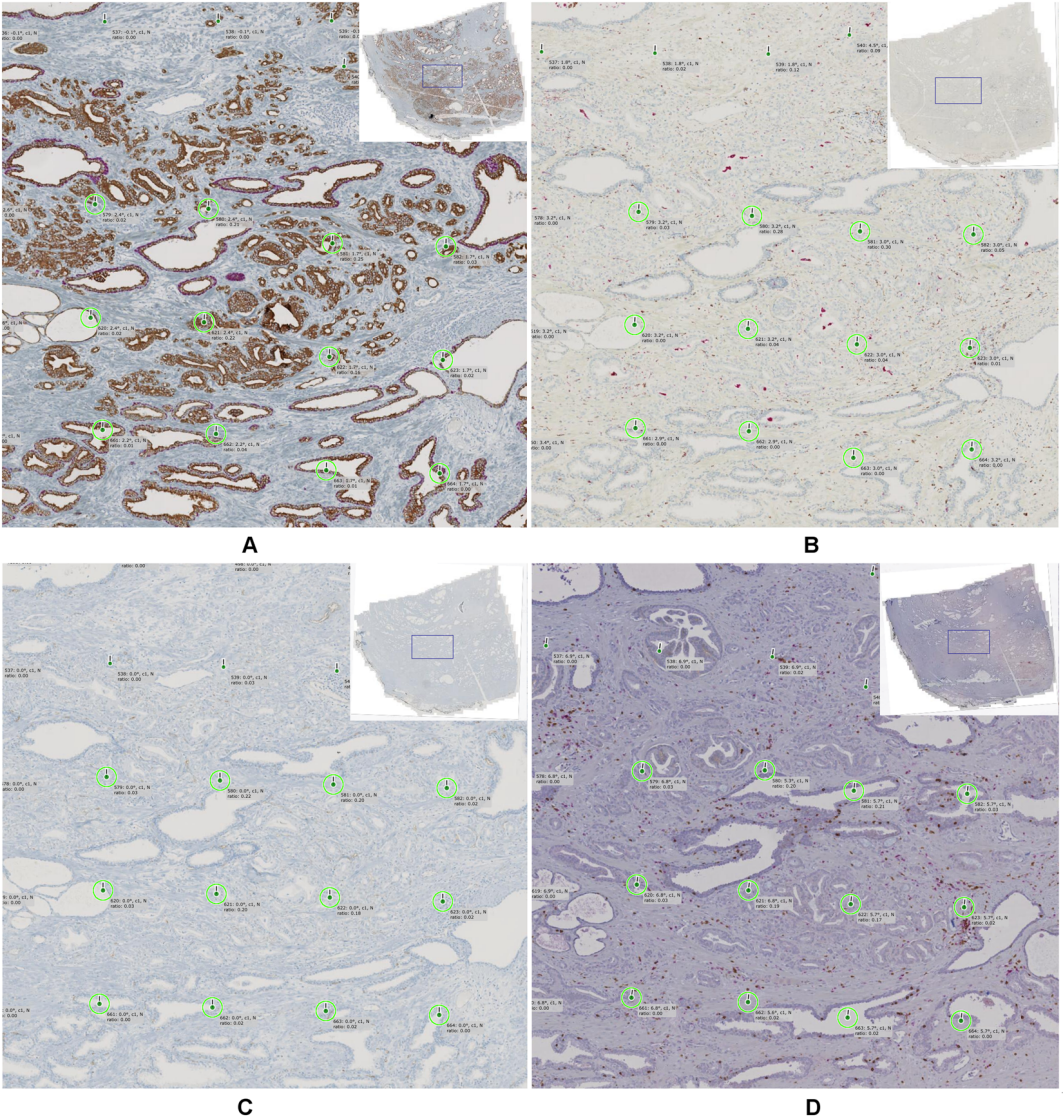
Co-registration example for a representative image region with four differently stained sections: (**A**) CK18/p63, (**B**) CD68/CD163, (**C**) CD34, and (**D**) CD3/CD8. Green circles highlight 12 corresponding landmarks which are used for spatial alignment. Here the tissue sections (**A**), (**B**), and (**C**) are relatively close, i.e. the gland structures are similar whereas section (**D**) seems to be more distant as the glandular structures are less similar.
  In the prostate cancer study a set of four serial tissue sections per patient was used. These sections were dual stained with quite dissimilar color and intensity ranges (Fig. 5). Thus, a multi-modal automatic co-registration approach was used (^17^ with local alignment module by microDimensions, Munich, Germany). With this approach, first, all tissue regions in all sections were automatically detected and a global rigid registration was performed for initial coarse alignment. To tackle the multi-modality problem, the images were normalized using a minimum intensity projection of the RGB image color channels and subsequent intensity inversion which allows using an intensity-based objective function (normalized cross-correlation) for optimizing the image alignment. After global alignment, co-registration was performed in a hierarchical fashion by splitting tissue regions into local patches and repeating the co-registration in sub-regions at multiple resolutions. Finally, landmarks encoding local rigid transformations were generated at the local patch region centers for the given target resolution (Fig. 5, green circles). For algorithm details see supplemental material, flow chart 3.
4. Data aggregation and quantification

The detected structures, their mutual relations, as well as the transformation parameters for spatial alignment are the basis for the aggregation and quantification step. The quality and the significance of the extracted data naturally depends on the quality of such previous steps, and thus, quality control is required prior to the quantification step. Next, a multitude of automated measurements of different complexity, and based on different types of object relations can be extracted. Object relations are characterized by the type, hierarchy level, and image section of the respective objects as well as by the type of combination, i.e. *within the same* or *between different* object types, hierarchy levels or image sections. This involves, for example, measurements of single structures of the same type and hierarchy level within the same section, such as cell morphology, cell staining intensity with respect to a specific marker, or region size, non-hierarchical relational characteristics between different object types within or between sections, such as the average distance of type 1 cells to their four nearest neighbors of type 2 cells. Also, hierarchical relational characteristics within or between sections, such as the number of nucleoli inside a nucleus, or the number of specific cell types inside the tumor region can be involved.

**Figure 6:**
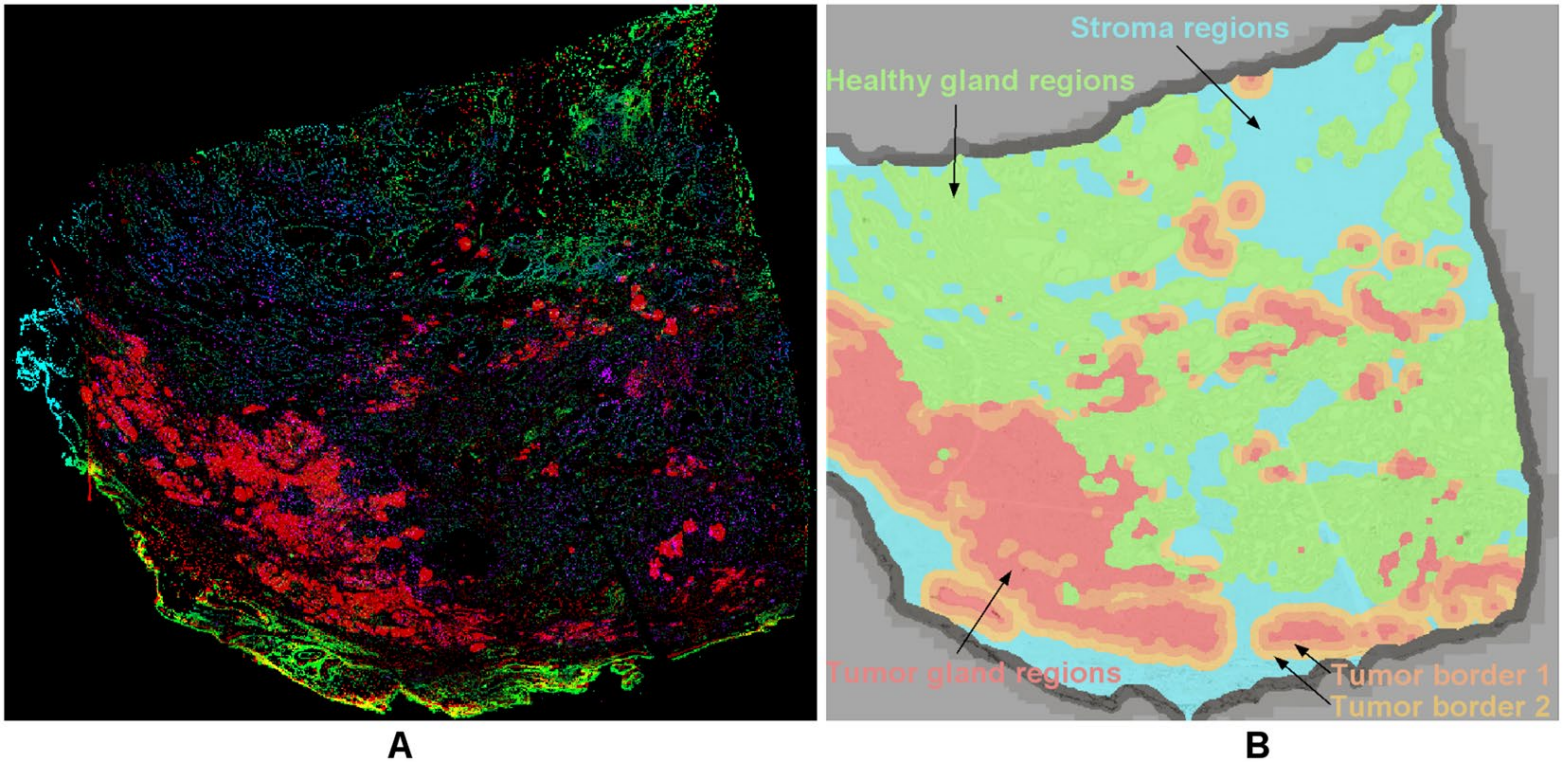
Heatmaps aggregating data on a lower spatial resolution. (**A**) Hyperspectral heatmap with 6 channels: average distance CD163(+) to CD68(+) macrophages (red) and vice versa (violet), CD34(+) vessel density (green), average distance of vessels to CD163(+) macrophages (blue), average distance of vessels to CD68(+) macrophages (green), tumor glands (area percentage, red), (**B**) heatmap with ROIs generated based on the structure-of-interest detection of healthy and cancerous glands and morphological operations on the heatmap level with tumor gland regions (red), tight tumor border 1 (dark orange), wider tumor border 2 (bright orange), healthy gland regions (green), stroma regions (light blue), and background or discarded border (gray).

A major challenge is to reduce the enormous number of possibilities to combine measurements for phene candidates in a meaningful way. For statistical reasons, the number of phene candidates needs to be limited compared to the number of cases in the study cohort. For efficient local quantification and visualization, measurements from different sections can be aggregated in lower spatial resolution representations (typically 100 microns per pixel), so called hyper-spectral heatmaps^39^. The term hyper-spectral refers to the large number of potential measurements, where each spectral channel of the heatmap represents one measurement (see Fig. 6A). Image analysis is used for detecting regions-of-interest (ROIs, see Fig. 6B) and meaningful structures in such hyper-spectral heatmap images, for example, unsupervised or knowledge-based segmentation and classification. Measures derived based on heatmap regions provide additional phene candidates.

In the prostate study we extracted measurements from all available stains. First, ROIs were defined based on the classified glands as regions containing primarily cancerous glands, regions containing healthy glands, and the remaining tissue regions, referred to as stroma (including stroma and other structures). Using morphological operations (opening, closing^40^) the identified regions were smoothed, and by dilating the tumor regions into the stroma we derived the close and wider tumor microenvironment with a width of 112.5 µm and 225 µm, respectively (Fig. 6B: light red and orange), as well as the tumor border defined as a region reaching equally far to the inside and to the outside of the tumor region (total width 112.5 µm, not shown in Fig. 6B). Next, we extracted quantitative information for each ROI, such as (I) region size, (II) absolute numbers of CD68(+), CD163(+), CD3(+), CD8(+), and CD34(+) cells per region, (III) average distances of all combinations of positive cells using 4 and 8 nearest neighboring cells (e.g., average distance of CD68(+) to their four nearest neighbors of type CD34(+)), (IV) distribution patterns of cancerous and healthy glands of different sizes^41^. Using region sizes (I) and absolute numbers (II) we also determined cell densities within each ROI. For algorithm details see supplemental material, flow chart 4.

### Data mining for Phene discovery

The raw measurements derived in the datafication workflow can be further processed and combined to enable more complex phenes. Relational combinations are used to provide all types of normalizations, such as area densities of marker-positive cells, percentages of marker-positive cells, and relations of positive cells of different types within the same or different ROIs. Moreover, other types of mathematical functions can be used to combine the extracted measurements in a meaningful way, where previous knowledge on expected relations may be integrated. However, note that the more complex the combinations of measurements the higher the risk of overfitting in the phene discovery step, and the more difficult the interpretation of the results. To further enrich the set of extracted phene candidates the image-based measurements can also be combined with other data types such as clinical measurements from blood samples or radiological tumor classification (TNM-classification), patient data (e.g., age, sex, weight), or gene expression data. Since phenotypes are functionally related to genotypes, enriching phene candidates with genomic or proteomic information offers potential to further increase the quality of their prognostic or predictive value. To facilitate the integration of multiple heterogeneous data sources to a single homogeneous dataset per individual, ontologies provide a common vocabulary and help bridging between different disciplines^42^. Moreover, Tissue Phenomics studies on prognostic and predictive phenes require knowledge on the disease progression, such as disease-free survival time (DFS), overall survival time (OS) or response to a certain type of therapy, which often resides in heterogeneous databases and needs to be linked to the images. Flexible bioinformatics software tools enable the cross-referencing using high-level scripting languages such as R^43,44^ or Cognition Network Language (see above).

The phene discovery process identifies a subset of phenes out of the pool of candidates which exhibit the best correlation to the clinical endpoints. The selection is performed by optimizing the quality of the solution for the biomedical question considering robustness while avoiding overfitting, and taking into account clinical applicability as well as economic constraints. To this end, machine learning techniques are used such as feature selection, supervised and unsupervised learning (e.g., clustering), and multi-parametric optimization of stratification or prediction models. The stratification quality typically is measured by Kaplan-Meier statistics such as the log-rank test significance and hazard ratio. As a core component of the Tissue Phenomics approach the optimization can loop back (Fig. 1) changing the parameters of the datafication process or creating variations of promising phene candidates to enable the evolution of potentially more discriminative phenes.

**Figure 7:**
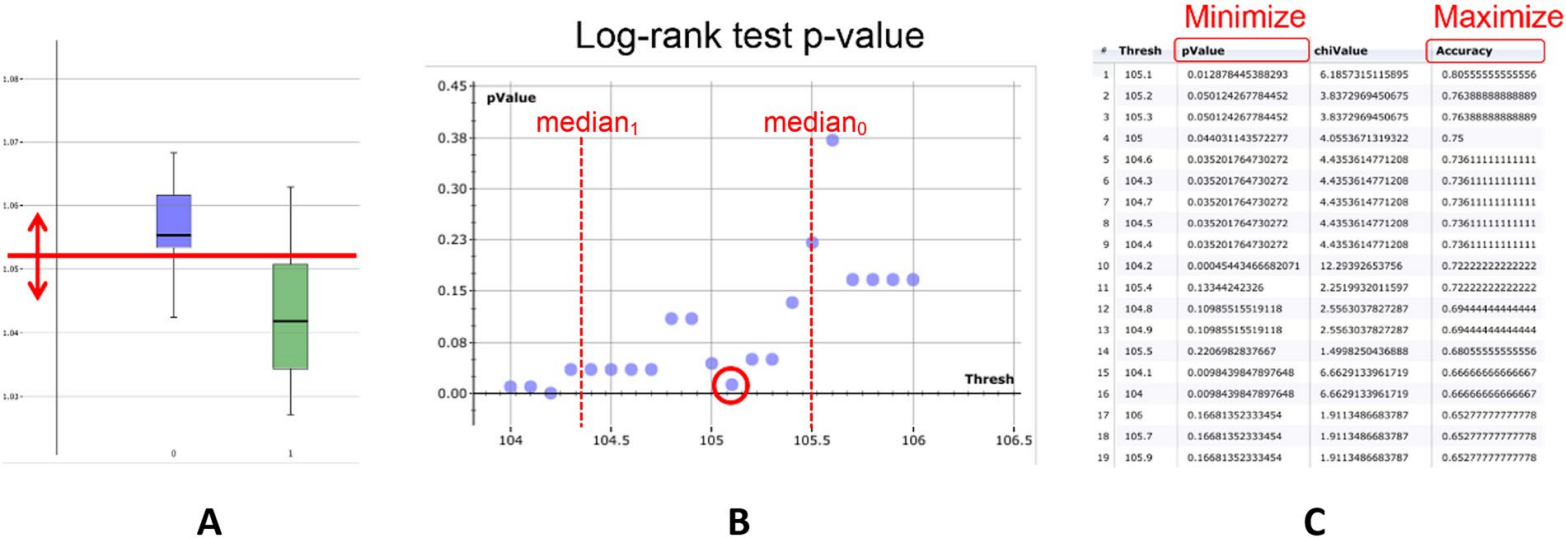
Phene candidate ranking based on Kaplan-Meier statistics (log-rank test) and classification accuracy. (**A**) Boxplot of one example phene candidate for the patient group showing tumor progression (green) and for the patient group with non-progression (blue), the bold horizontal line inside each box represents the group median. For threshold optimization values in the range between the medians are tested (red horizontal line). (**B**) Log-rank test p-values for different thresholds where the relevant local minimum is marked (red circle). (**C**) Corresponding list of p-values and accuracies for different thresholds as used for optimization.

Phene candidate processing in the prostate cancer study involved all types of relational combinations within the same or different ROIs and sections. Additional data sources were not included, since one particular goal of the study was to provide improved prognostic markers that are purely image-based and complement the standard features used for prognosis by pathologists (e.g., PSA value, pT staging, Gleason score, age). Phene discovery was done by ranking the extracted phene candidates according to their relevance by systematically testing the stratification performance regarding the clinical endpoint disease-free survival time (DFS) with the event PSA recurrence (indicating tumor progression). For each phene candidate we optimized a threshold for classifying patients regarding the event (tumor progression/non-progression) by iterating the threshold values in the interval between the medians of the two event classes (using the known patient outcome data). In each iteration a log-rank test was performed and the classification accuracy was computed as the fraction of correctly classified patients. The threshold minimizing the log-rank test p-value while maximizing the classification accuracy was selected as the optimum (see, e.g. refs^45^). Finally, all phene candidates were ranked regarding their optimal classification accuracies and p-values. The top-ranking phenes with their optimized thresholds provided basic uni-variate classifiers (Fig. 7).

**Figure 8:**
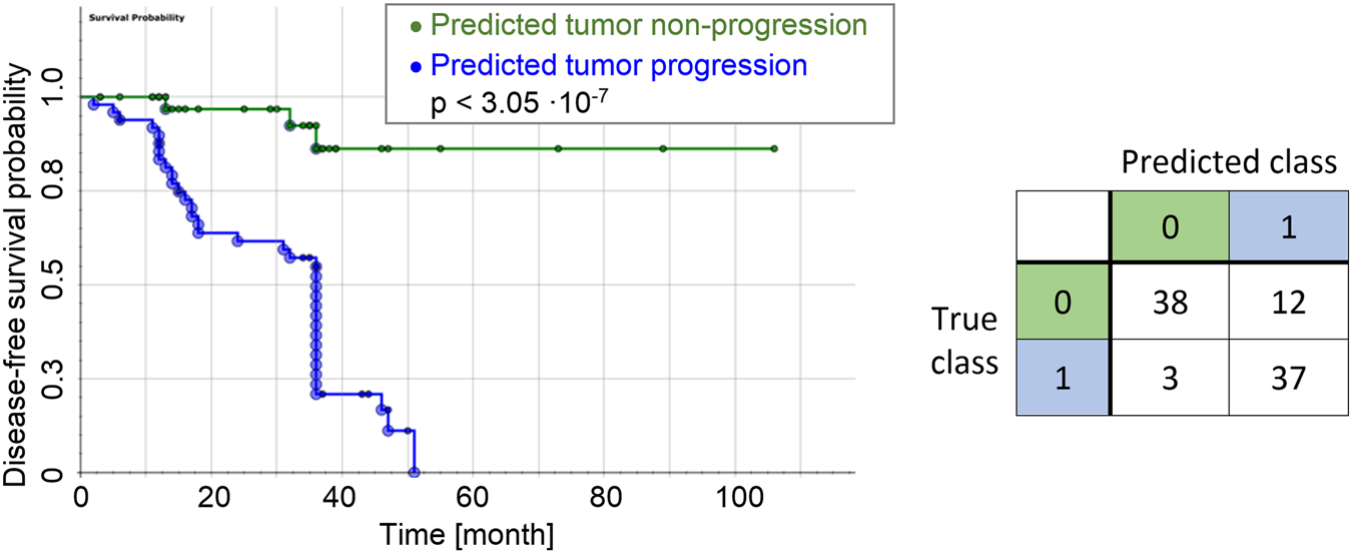
(Left) Kaplan-Meier plot for the prediction based on the top-ranking phene candidate *ratio of CD8(*+*) and CD34(*+*) in tumor microenvironment* using a leave-one-out cross validation scheme (log-rank test on aggregated prediction p < 3.05·10^−7^, prediction accuracy 83.3%). (Right) Confusion matrix where rows provide the true class and columns the predicted class (0: non-progression, 1: progression). (Note that for some patients with DFS times of up to 36 month the exact disease recurrence dates were not available and only the approximated DFS time of 36 month could be used, leading to the event aggregation at 36 month in the plot).

However, given the large number of around 700 phene candidates and the limited number of *n* = 90 patients this method is prone to overfitting. Therefore, we performed leave-one-out cross validation in the threshold optimization and ranking procedure, where in *n* iterations *n* − *1* samples were used for training and prediction was done on the one left-out sample per iteration, thereby successively predicting all cases. This procedure resulted in more realistic performance estimates and favored phenes that are robust with respect to small alterations of the data set. Therefore, the number of occurrences at the top of the per-fold ranked lists was quantified for each candidate over all cross validation folds, providing an estimate of the candidate’s robustness. Phene ranks were assigned based on the classification accuracy on the test folds, aggregated over all cross validation folds per phene candidate as well as on the robustness in terms of top-5 occurrences. Based on the final ranking we selected the top *k* phenes from the ranked phene list. For example, Fig. 8 shows the Kaplan-Meier plot for the top-ranking phene applied as a uni-variate classifier using the average optimal threshold over all cross validation folds. To find the number of statistically relevant candidates *k*, we performed feature selection based on multiple-testing correction on the p-values derived from the log-rank test on the cross-validated aggregated predictions. To this end, first, poor-performing candidates with cross-validated accuracies ≤50% were removed from the list of candidates. Next, we performed multiple-testing correction using Bonferroni correction, a family-wise error rate method (FWER^46^), and a method controlling the false discovery rate (FDR^47^). Since Bonferroni correction assumes independence of the feature values we first applied a correlation filter which removes strongly correlated features in an iterative procedure (maximum allowed absolute correlation MAC < 0.75). Using the corrected significance levels α_corr_, smaller sets of relevant phenes with p-values < α_corr_ were obtained. For example, using the Holm-Bonferroni correction^46^ 20 significant phenes were identified (supplemental Table S3).

To further reduce the number of relevant phene candidates we performed feature subset selection on the relevant phenes in supplemental Table S3 using the supervised classification method CART (Classification and regression trees^48^). CART training builds a decision tree of a given depth, where for each individual branch of the tree the feature providing the best class separation is selected from the list of features. Thus, if the number of decisions in the tree is smaller than the number of available features, the classifier performs automatic feature subset selection. In a leave-one-out cross validation procedure we aggregated the frequencies of phenes being selected by CART training over all cross validation folds. Table 2 provides the resulting list of 8 phenes that have been selected in at least one cross validation fold along with the previously determined average threshold, performance and the selection count (for interpretation of the phene names see Table 3). The relevance of the selected top-ranking phenes was additionally approved by Cox regression analysis (supplemental Table S4). For algorithm details see supplemental material, flow chart 5.

**Table 2:** Subset of significant phenes resulting from feature selection after multiple-testing correction (supplemental Table S3) using CART in a cross validation scheme. Column **Phene** provides a short definition (for interpretation of the phene names see table panel B), which together with the operator **OP** and the threshold **THR** defines the model for classifying patients as tumor non-progression (distances are given in µm). **ACC** provides the cross-validated accuracy and **NUM** represents the frequency each phene candidate has been selected by the CART classifier over all cross validation folds. For interpretation of the phene names see Table 3.

	Model: tumor non-progression if			ACC	P-VAL	NUM
	Phene	OP	THR			
1	RATIO #**CD8**_*border1* TO #**CD34**_*border1*	>=	0.10	0.83	3.05e-007	86
2	DIST **CD68** TO **CD34** IN *nonIntact*	>=	75.70	0.82	2.89e-008	90
3	CORR Haralick on intact & non-intact glands	<	0.09	0.82	4.68e-006	85
4	RATIO #**CD8**_*nonIntact* TO #**CD163**_*nonIntact*	>=	0.40	0.74	0.00021	4
5	DIST **CD3** TO **CD8** IN *border2*	<	99.45	0.73	9.71e-005	2
6	RATIO #**CD68**_*border2* TO #**CD34**_*border1*	>=	0.04	0.76	1.84e-005	1
7	DIST **CD3** TO **CD8** IN *innerBorder*	<	98.69	0.74	0.00047	1
8	RATIO #**CD8**_*ws* TO #**CD3**_*ws*	>=	0.73	0.72	7.80e-005	1

**Table 3:** Naming conventions explaining how to interpret phene names.

	Operation	Argument 1	Separator	Argument 2	Separator	Location
Examples	RATIO	#**CD8**_*border2*	TO	#**CD163**_*border1*		
	DIST	**CD163**	TO	**CD8**	IN	*intact*
	DENSITY	**CD34**			IN	*stroma*
**Operations**				**Locations**		
• RATIO: ratio of arguments				• intact: healthy gland regions		
• DIST: average distance from object of type Argument 1 to its four nearest neighbors of type Argument 2				• *non-intact*: cancerous gland regions		
• DENSITY: number of pixels of type Argument 1 divided by region size (in number of pixels) of region Location				• *stroma*: stroma regions		
				• *innerBorder*: tumor border reaching to the inside and to the outside (56 µm in each direction)		
• CORR Haralick: gland co-occurrence features quantifying the joint appearance of different gland types				• *border1*: tight tumor border (112.5 µm)		
				• *border2*: wider tumor border (225 µm)		
				• *ws*: whole slide including all regions excluding non-tissue background		
**Arguments**						
• **biomarker** for which the considered objects are positive						
• **#biomarker**_*location*: absolute number of objects within the given region (see location)						

**Figure 9:**
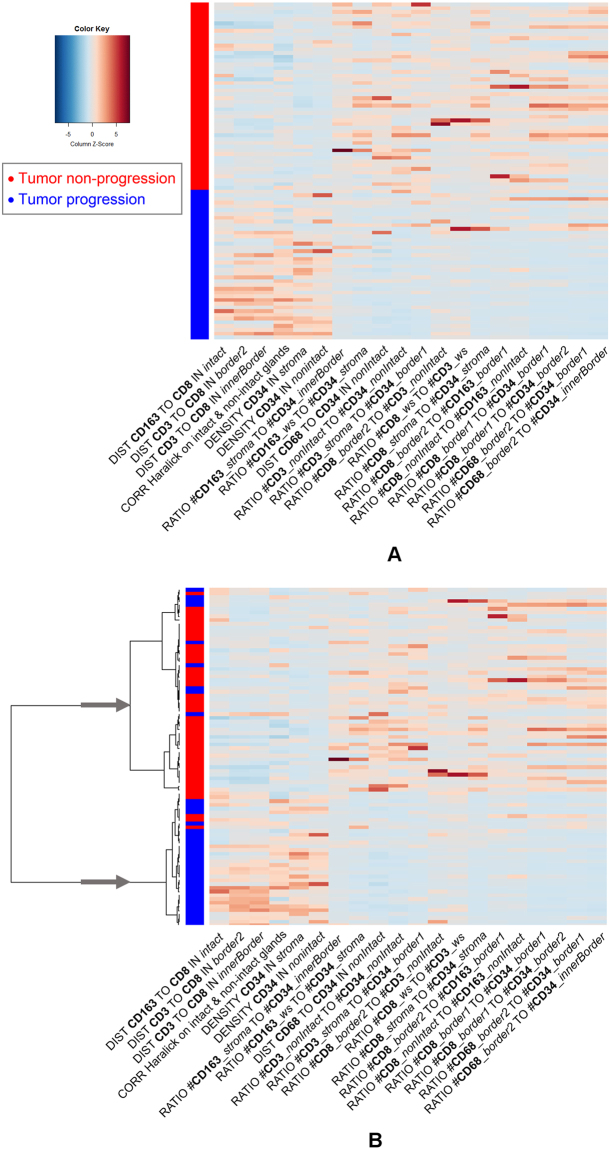
Heatmaps of 20 phene candidates with significant log-rank test results for the stratification of patients with respect to tumor progression (blue) and tumor non-progression (red) using Holm-Bonferroni correction. (**A**) Rows sorted by clinical endpoint, (**B**) rows sorted by clustering, the arrows mark the two main clusters used for classification. (For interpretation of the phene names see Table 3).

The selected phene sets were employed for multi-variate stratification using supervised or unsupervised learning methods (see Sect. Results below). Figure 9, for example, shows the result of unsupervised hierarchical clustering on the 20 selected phenes (after z-score normalization) similar as used in genomics data analysis^49^.

To test the significance of the highest yielded accuracies and p-values reflecting the importance of the top-ranking phene candidates, we performed permutation tests. Feature vectors (i.e. the individual phene candidate values) of all patients were randomly shuffled against their clinical outcome data (DFS, event) and the phene candidate ranking was performed as described above. Shuffling and ranking was repeated 100 times generating a distribution of top-ranking accuracies and p-values for the permuted data sets (mean_accuracy_ = 0.68, std dev_accuracy_ = 0.02, mean_pValue_ = 0.01, std dev_pValue_ = 0.02). The approximated probability of yielding similar or higher accuracies for the permuted set compared to the unpermuted data turned out to be zero for most of the selected 20 phenes (15 out of 20) emphasizing the significance of the top-ranking phenes (permutation test p-value < 0.05 for 13 top ranking phenes). (Note that we did not use cross validation when generating the permutation distribution.) For a general summary of feature selection methods in the context of Genomics and Proteomics see, e.g. ref.^50^.

### Phene validation

Once the best-performing phenes are selected and before they can be considered for clinical evaluation, a comprehensive validation of the discovered phenes is inevitable. It is particularly important to prove the relevance of such phenes for independent patient cohorts from different sites with potentially different cutting and staining protocols, as well as different slide scanning routines and scanners. Besides, the discovered phenes need to be interpreted and validated using domain knowledge from other studies. Finally, dedicated clinical studies can be performed to prove the relevance and significance of the identified tissue-based prognostic phenes.

In the prostate study, phene validation so far has been addressed by literature research where we identified studies supporting the found hypotheses (see section Discussion). However, a validation study on unseen data from a separate clinical site needs to be performed as a next step. In the light of slow prostate cancer progression, a prospective study using the disease-free survival as a clinical endpoint is challenging in terms of study time and resources.

### Data availability

The datasets generated during and/or analyzed during the current study are not publicly available due to ongoing work on the data analysis but are available from the corresponding author on reasonable request.

### Ethical approval

The current study was approved by the Ethics Committee of the Medical Faculty of the Ludwig Maximilians University (LMU), Munich, Germany, and the need for consent was waived by the institutional ethics committee (Eu Nr 099-14). Prostate cancer specimens from patients that underwent surgical resection at the LMU were drawn from the archives of the Institute of Pathology. All tumor tissue used was leftover material that had initially been collected for histopathological diagnostics and all diagnostic procedures had already been fully completed when samples were retrieved for the study. All patient data were fully anonymized, and the study was performed according to the standards set in the Declaration of Helsinki 1975. Authors were blinded for clinical information during experimental analysis.

## Experimental Results

### Segmentation and co-registration performance

**Figure 10:**
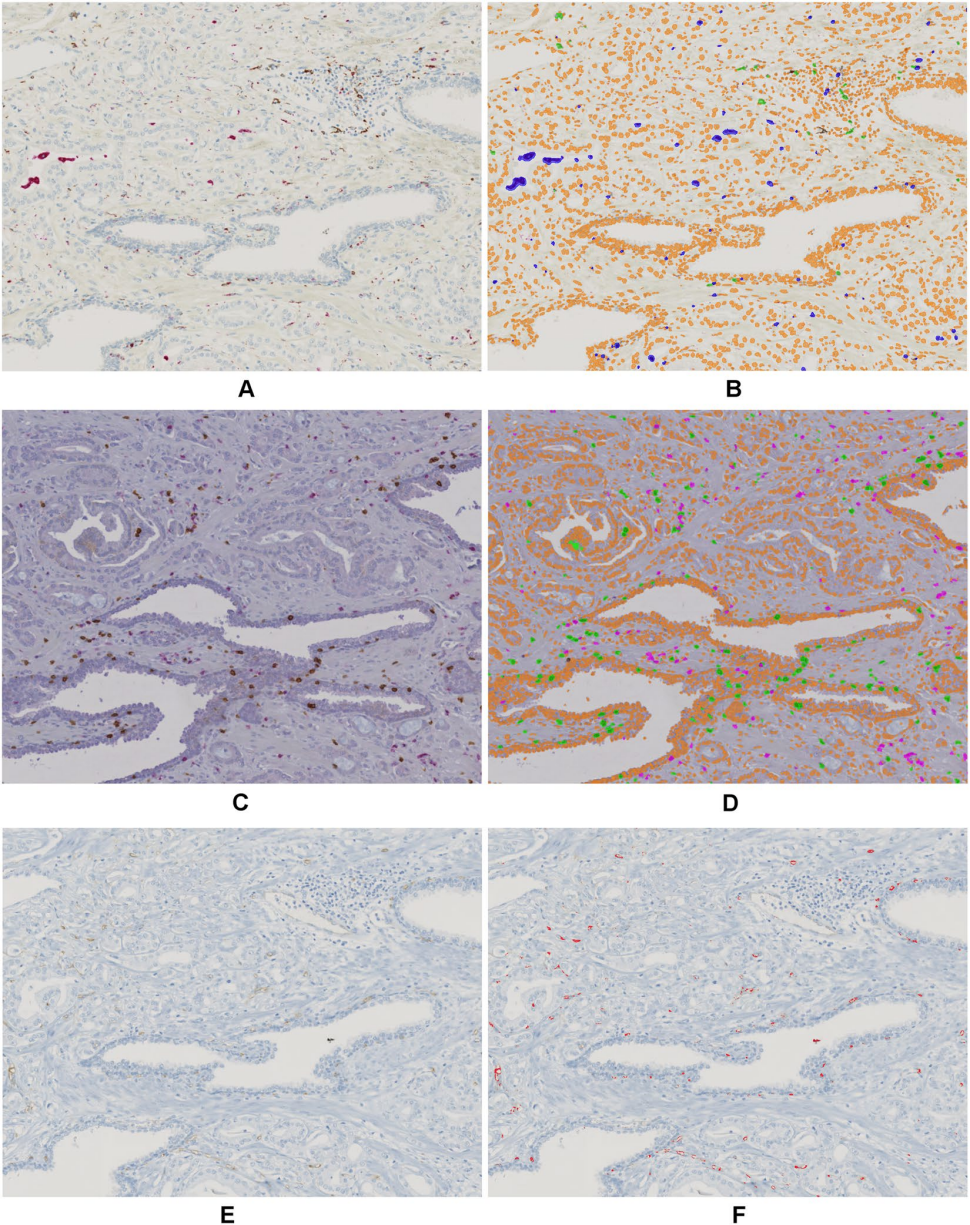
Automatic segmentation results with detection of cells, cell nuclei, and vessel structures. (**A**) CD68/CD163 raw image and (**B**) segmentation result with CD68(+) M1 macrophages (blue overlay), CD163(+) M2 macrophages (green overlay), and negative cell nuclei (orange overlay), (**C**) CD3/CD8 raw image and (**D**) segmentation result with CD3(+)CD8(−) T-cells (magenta overlay), CD8(+) cytotoxic T-cells (green overlay), and negative cell nuclei (orange overlay), (**E**) CD34 raw image and (**F**) segmentation result with vessel structures (red overlay).

The accuracy of the cell segmentation has been evaluated on a large set of different types of images elsewhere^37^, and it turned out that the approach yields an average nucleus segmentation accuracy of 78% measured over different IHC stains. For the images analyzed in this work we performed qualitative assessment of the segmentation accuracy by visual inspection. In all inspected cases a sufficiently high segmentation accuracy was observed (see Fig. 10A–D). The accuracy of the gland and vessel segmentation was approved by visual inspection as well. For exemplary results see Fig. 4A,C and Fig. 10E,F, respectively.

For a performance evaluation of the co-registration approach see^17^. Since the quality of the co-registration naturally depends on the integrity of each tissue section, as well as on the cutting distance of the physical sections we inspected all co-registration results and corrected errors where necessary (whole slide flips or larger displacements). Overall, such manual corrections were required only for three out of 90 processed cases.

### Phene discovery

#### Phene ranking and subset selection

Using the phene ranking and selection approach described above we obtained a list of 20 significant phene candidates (supplemental Table S3) by applying the Holm-Bonferroni-corrected significance level α = 0.05 after filtering for extremely low accuracies (<50%) and strongly correlated candidates (r > 0.75). In addition, we obtained a smaller subset of 8 phene candidates (Table 2) using CART feature selection.

#### Patient stratification performance

**Figure 11:**
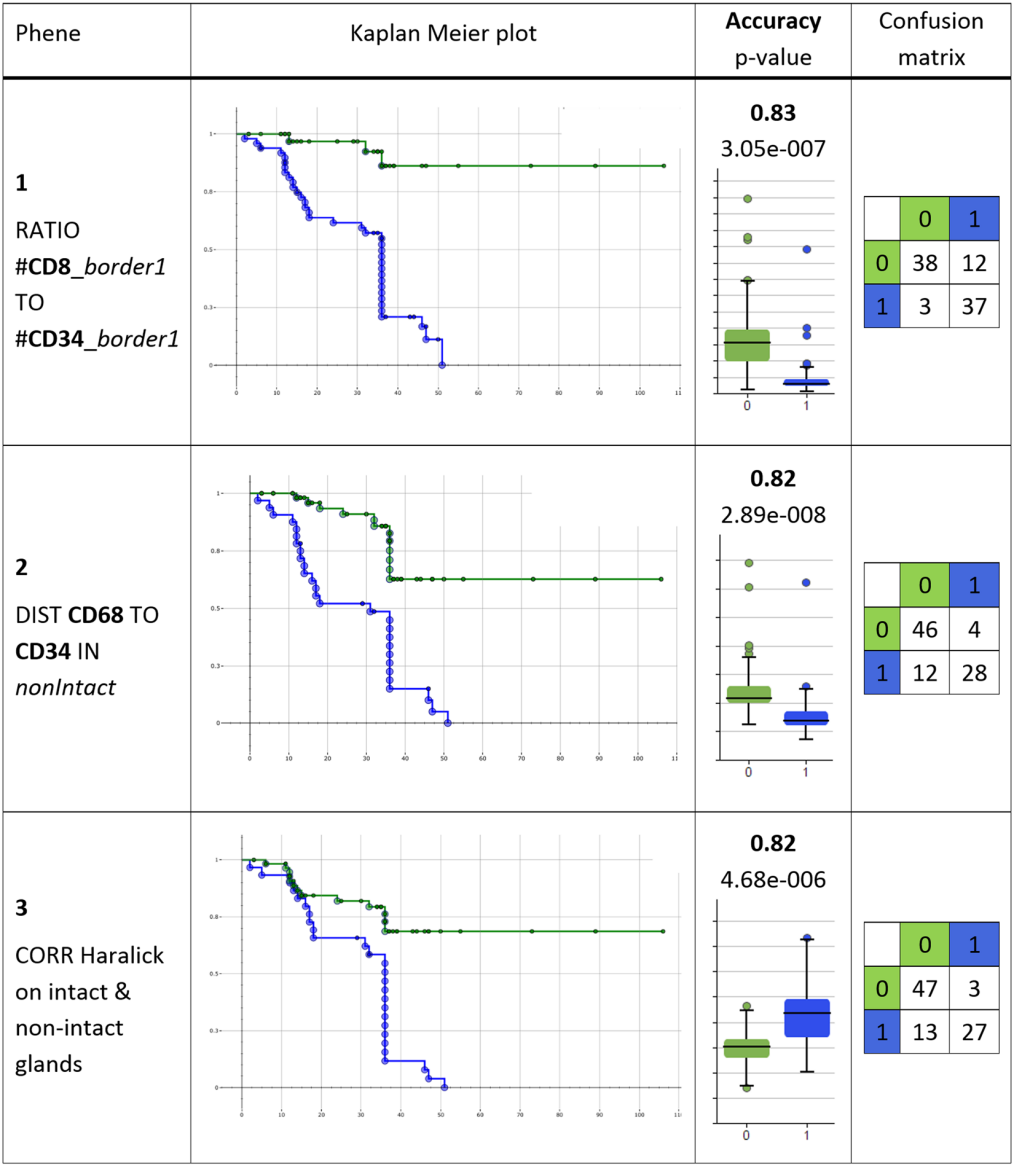
Result for uni-variate stratification with cross validation using the top-3-ranking significant phenes in Table 2. The Kaplan-Meier plots show groups “non-progression” (green) versus “progression” (blue) as predicted, with the events corresponding to true tumor progression state. X-axis: disease-free survival time (DFS) in month, y-axis: disease-free survival probability. The provided accuracies and p-values are obtained by aggregating the test set predictions in a leave-one-out cross validation. The box plots visualize the respective distributions for each phene. In the confusion matrices rows provide the true class and columns the predicted class (0: non-progression, 1: progression). (Note that for some patients with DFS times of up to 36 month the exact disease recurrence dates were not available and only the approximated DFS time of 36 month could be used, leading to the event aggregation at 36 month in the plot).

Uni-variate stratification: The most direct approach to apply the selected phenes for patient stratification is using uni-variate classification, that is, patients are stratified based on a single phene with a threshold optimized on the training set of patients with known clinical outcome (as described above). Figure 11 provides the Kaplan-Meier plots along with the accuracies and confusion matrices for uni-variate stratification using leave-one-out cross validation for the top-3-ranking phenes given in Table 2 (see supplemental Fig. S4 for all corresponding Kaplan-Meier plots). As can be seen in the first row of Fig. 11 the top-ranking phene “RATIO #**CD8**_*border1* TO #**CD34**_*border1*” yields a highly sensitive stratification as shown by the Kaplan-Meier plot, with only 3 false negative and 12 false positive predictions (for interpretation of the phene names see Table 3).

Multi-variate stratification: More complex models for patient stratification combine multiple phenes, for example, using unsupervised or supervised classifiers. Here we compared the stratification performance for one unsupervised and six different supervised classification methods using the selected sets of significant phenes as presented in Table 2 and supplemental Table S3. In particular, we used hierarchical clustering^51^, Bayes classifiers, CART classifiers (depth = 2)^48^, k-nearest neighbor classifiers (KNN with k = 5), linear predictor models, and support vector machines^52^ with linear and radial basis function kernels.

**Figure 12:**
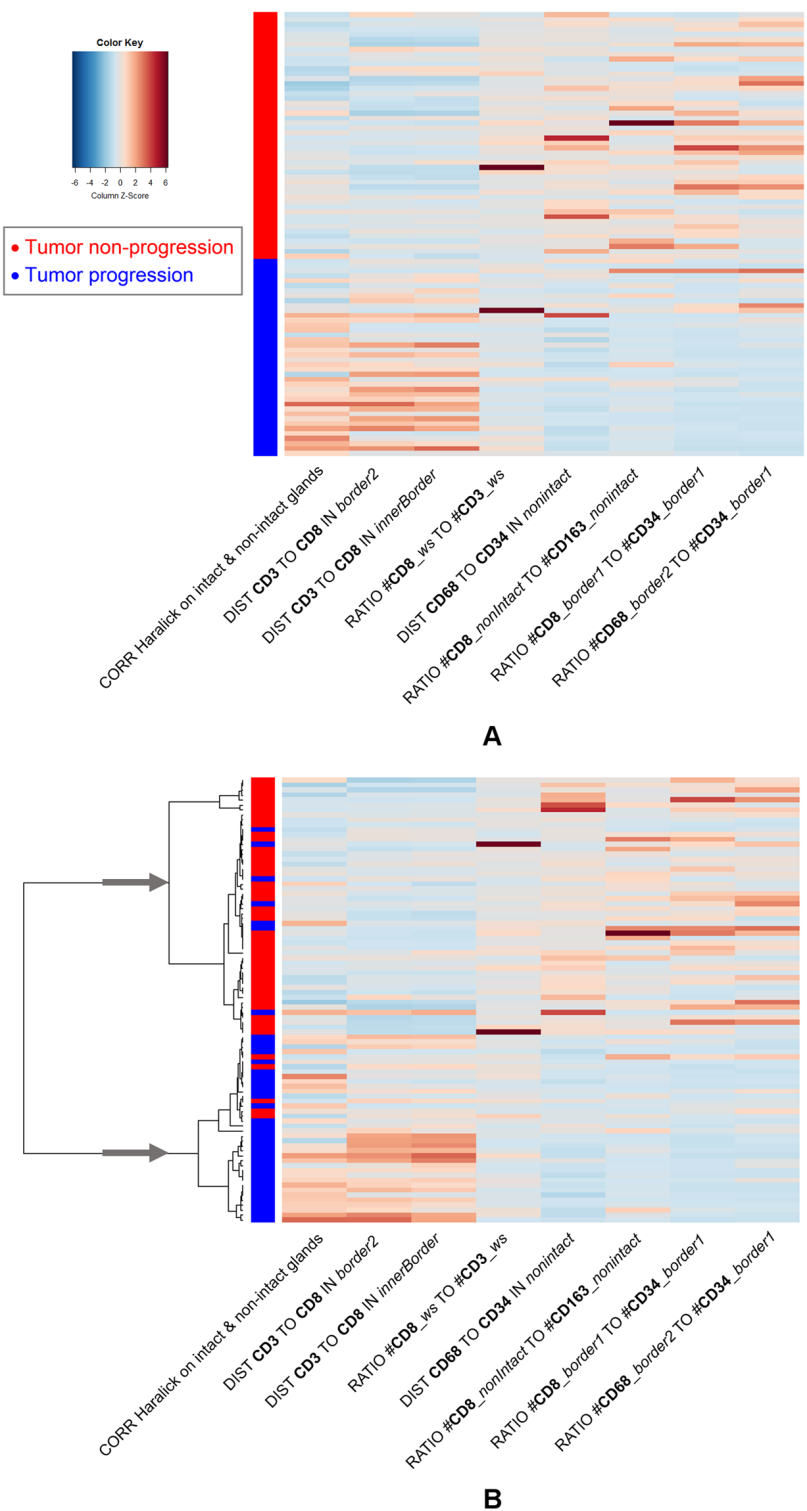
Heatmaps of 8 phene candidates with significant log-rank test results for the stratification of patients with respect to tumor progression (blue) and non-progression (red) using Holm-Bonferroni correction and CART-based feature selection. (**A**) Rows sorted by clinical endpoint, (**B**) rows sorted by hierarchical clustering, the arrows mark the two main clusters used for classification. (For interpretation of the phene names see Table 3).

In case of the unsupervised hierarchical clustering we did not use cross validation since clustering operates on the feature values only without considering the class labels. To obtain predictions the clustering tree resulting from stepwise bottom-up merging of similar clusters (distance measure: 1-*Pearson correlation*^48^) was cut after the top-most branching (see dendrograms in Figs 9 and 12), thereby assigning each patient to one of the two main clusters. The obtained clustering corresponds very well with the true endpoint tumor progression/non-progression as can be seen in Figs 9B and 12B, and yields overall accuracies of 84% (log-rank test p < 1.25e-007) for the phene set identified by Holm-Bonferroni correction (supplemental Table S3), and 87% (log-rank test p < 2.14e-008) for the phene set from subsequent CART feature selection (Table 2). Note that we obtained higher accuracies for the smaller phene set, suggesting that using more candidates does not provide additional discriminatory power.

**Figure 13:**
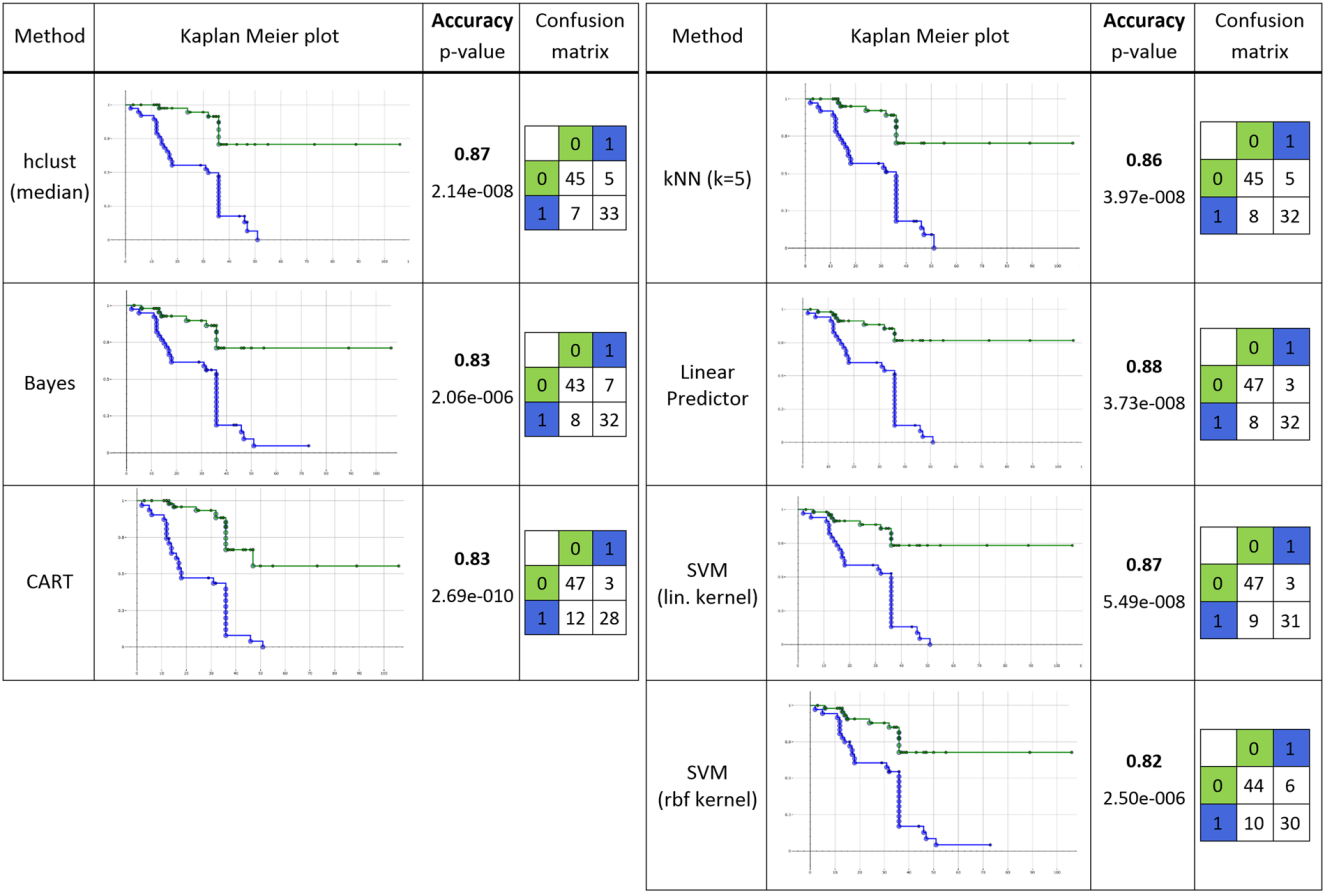
Performance evaluation of multi-variate stratification using different types of classifiers with the subset of 8 significant phenes selected using the FWER method of Holm-Bonferroni followed by CART-feature selection (Table 2). The Kaplan-Meier plots show groups “non-progression” (green) versus “progression” (blue) as predicted, with the events corresponding to true tumor progression. X-axis: disease-free survival time (DFS) in month, y-axis: disease-free survival probability. In the confusion matrices rows provide the true class and columns the predicted class (0: non-progression, 1: progression). (Note that for some patients with DFS times of up to 36 month the exact disease recurrence dates were not available and only the approximated DFS time of 36 month could be used, leading to the event aggregation at 36 month in the plot.

All supervised classification methods have been tested in a leave-one-out cross validation framework and we computed the Kaplan-Meier statistics (log-rank test) for the resulting aggregated set of predictions. The classification results as well as the Kaplan-Meier plots and statistics for all studied supervised classifiers are provided in Fig. 13 and supplemental Figure S5 along with the result of the unsupervised clustering method. It turned out that for the multi-variate stratification we generally yield higher accuracies of 81% to 88% than for the uni-variate stratification with accuracies of 80% to 84%, and highly significant log-rank test p-values for all tested classifiers (2.69e-010 to 2.46e-005). For the larger set of 20 phenes the top-performing classifiers were the k-nearest neighbor classifier (k = 5) (88%) and support vector machines, where the linear kernel (87%) performed slightly better than the radial basis function (rbf) kernel (86%). For the smaller set of 8 phenes the linear predictor performed best (88%) closely followed by support vector machines with linear kernel (87%) and the unsupervised hierarchical clustering (87%). Thus, the larger set did not provide additional relevant information as compared to the reduced set which offered sufficient discriminatory power for yielding similar accuracies. However, further reducing the number of selected phenes led to a decrease of accuracies (data not shown). In conclusion, we found the reduced set of 8 phenes selected in the described multi-stage procedure provided the most relevant phenes yielding the highest accuracies by multi-variate stratification. Among the top-performing classifiers the support vector machines with linear kernel performed very well on both phene sets.

## Discussion

For many decades, the microscopic investigation of tumor tissue has been the basis and gold standard for cancer diagnosis and treatment, putting the pathologist close to patient care by giving essential guidance for treatment decisions. Extracting information from tissue in histopathology often is still predominantly a manual process performed by expert pathologists. This process to a certain degree is subjective and depends on the personal experience of each pathologist, it is rather qualitative than quantitative, and of limited repeatability as the considerable variability in histological grading among pathologists exemplifies. Moreover, conventional histopathology currently does not allow systematic extraction of the rich information residing in tissue sections. The histological section represents the complex phenotype in a solid way and as Caie *et al*^53^. pointed out, the entirety of DNA, RNA, proteome and metabolome are all blended into a topologically ordered and preserved morphological integrity. Therefore, the transition from a more descriptive to a strictly quantitative discipline being the core objective of modern histopathology, is clearly worthwhile to pursue. This will allow the extraction of large amounts of tissue-derived data from many patients, enabling the creation of unprecedented knowledge through statistical evaluations and improving patient treatment.

Conventional approaches such as H&E-based morphological assessment have been increasingly complemented by proteomic and genetic methods, mainly through immunohistochemistry (IHC) and *in situ* hybridization. Genomic studies, although not providing microscopic spatial resolution, started to add value by furnishing rich genetic fingerprints. Several successful attempts have been developed to understand cancer determinants in tissue-based genomics with platform technologies such as qPCR and next-generation sequencing. The importance of genomics in oncology is natural and appears evident as cancer is a genetic disease driven by specific genetic mutations. In a number of cases correlations between gene mutations and diseases have been identified and associated diagnostic tests have been established^54,55^. However, today only limited parts of available data are of known clinical relevance and are actually used for diagnostic or therapeutic decision-making. Tumors are known to be heterogeneous and many different mutations or wild-type gene expression-related features occur that might affect patient outcome. Also, the tumor and its microenvironment are characterized by spatial patterns, such as the arrangement of cells involved in the interaction of the immune system with the tumor, which cannot be adequately characterized by genomic approaches yet is known to be highly relevant for prognosis^56^. Even before the first human genome was fully sequenced, it became obvious that hopes and salvation expectations projected onto the emerging field of genomics would be challenging to fulfill.

The concept of the phene, coined a century ago, gained novel significance after being overshadowed by genetic approaches for years. An independent discipline named *phenomics* was postulated to help elucidate the physiological hierarchies leading from genetic traits to clinical endpoints^57^. A decade later, it was understood that the systematic study of phenotypes in relevant biological contexts would be “critically important to provide traction for biomedical advances in the post-genomic era”^58^. Comprehensive phenotyping addresses complexity on different scales: From transcriptomics and epigenomics over proteomics to high-throughput assays of cell cultures biological entities of increasing hierarchical complexity are assessed systematically^59^. However, these approaches are compromised by methodological challenges. Performing phenomics on a large scale is an intrinsically multi-dimensional problem, because the phenome is highly dynamic. It is influenced by a multitude of factors from post-translational modifications to high-level environmental stimuli. Approaches like proteomics, capturing temporally highly focused snapshots of multifactorial biological processes, are limited in their transferability and significance. In general, the gigantic molecular network that acts between the genome and the unfolding of tissue structures with all their properties is still not fully understood. Tissue Phenomics provides a systematic way of knowledge discovery thereby contributing to fill this knowledge gap.

In this work, we presented a comprehensive introduction as well as a proof-of-principle application of the Tissue Phenomics approach for automatic phene discovery in prostate cancer. In particular, we demonstrated that tissue phenes with high prognostic value can be identified fully automatically based on few general assumptions such as the importance of immune cells for cancer evolution. Importantly, such phenes rely on mathematical relations of spatial distribution patterns of key immune players that would hardly have been possible to discover by manual inspection due to the large amount and high dimensionality of the data. Also, since the discovered phenes represent relations of objects from different IHC stains and measures derived by statistical evaluation within regions-of-interest which are not necessarily visible in every stain, such phenes typically cannot be seen directly by looking at the raw images. Using advanced image analysis methods for co-registration and segmentation we derived a large set of potential phenes. We ranked the phene candidates and selected a subset of phenes showing the highest prognostic value regarding tumor progression based on sound statistical evaluation. The selected phenes yield high accuracies of up to 83% regarding the uni-variate stratification of patients into disease progression groups. Although such phenes reflect nontrivial relationships of different players of the immune response they are interpretable and can be related to existing knowledge in the field. Combining the discovered phenes using machine learning techniques yields even higher accuracies of up to 88%. However, interpretation of the biological meaning is less intuitive for the resulting multi-variate models. An exception are decision trees where the outcome is obtained by iterating through a tree of subsequent decision rules similar as in many medical decision making processes by clinicians. Thus, such classifiers might be easiest to communicate and to be accepted in clinical practice.

The most promising phenes identified in this study have been investigated regarding their biological plausibility and turned out to agree well with previous findings as described in literature. For example, the top-ranking phene “RATIO #CD8_*border1* TO #CD34_*border1*” (Table 2) suggests that a high ratio of areas of CD8(+) cells and CD34(+) microvessels in the tight tumor border are correlated with a good prognosis and long-term disease-free survival (for interpretation of the phene names see Table 3). This corresponds to high densities of CD8(+) cells and/or low microvessel densities, which as separate properties have been shown to be associated with good prognosis in prostate cancer in the literature: The hypothesis that CD34-based microvessel density is a significant predictive and prognostic factor is widely accepted and confirmed by various studies^60,61^, while the prognostic properties of tumor-infiltrating lymphocytes are more differentiated. Studies on general TIL density do not show a clear trend on whether high or low densities are beneficial^62–66^ since different subpopulations of effector and regulatory T lymphocytes are involved. However, high densities of tumor-infiltrating CD8(+) lymphocytes seem to be beneficial, being constructive mediators of an effective antitumor response^67^. The second-best phene “DIST CD68 TO CD34 IN *nonIntact*” which is also closely related to the 6^th^-ranked “RATIO #CD68_*border2* TO #CD34_*border1”* as well includes the microvessel density in the tumor region, stating that larger distance of CD68(+) macrophages to microvessels or a high density of CD68(+) macrophages together with a low microvessel density point to a good prognosis. Again, a low microvessel density seems to be beneficial as well as a higher density of CD68(+) macrophages. For tumor-associated macrophages typically the CD68(+) M1-polarized phenotype is associated with tumor-suppressing properties indicating that a higher density of this population compared to the M2 type is beneficial^65,68^. On the contrary, high densities of CD163(+) M2-polarized macrophages are known to exhibit tumor-promoting behavior^68–71^. This corresponds well to the 4^th^-ranked phene “RATIO #CD8_*nonIntact* TO #CD163_*nonIntact”* where a high density of CD8(+) and importantly a low density CD163(+) relate to a good prognosis. Finally, also the mixture pattern of differently-sized non-cancerous and cancerous glands provides prognostic value as shown by the 3^rd^-ranked phene “CORR Haralick on intact & non-intact glands”. Here, a low linear dependency between glands of different types relates to cases without tumor progression. This to a certain degree reflects and refines the criteria used for Gleason scoring by systematically quantifying glandular patterns.

Current limitations of the prostate study include the moderate size of the study population (90 patients), the lack of an independent validation cohort from a different clinical site, as well as the limited length of the follow-up times for certain patients. In future work we are aiming to improve on these limitations, in particular, to test our findings on an additional patient cohort. Also, we will validate the prognostic phenes we identified in whole slide images from resected prostate tissue in fine needle biopsies. If similar patterns of the immunological landscape are observed also in needle biopsies Tissue Phenomics will directly improve critical treatment decision in prostate cancer patients, for example, avoiding an unnecessary surgery, particularly in the case of intermediate risk patients. Finally, also in the context of immunotherapies Tissue Phenomics offers large potential to better stratify patients for the treatment options currently available for prostate cancer^72^.

## Electronic supplementary material

Supplementary Material
