# Supplementary material for: Tissue Phenomics for prognostic biomarker discovery in low- and intermediate-risk prostate cancer
Source: Sci Rep. 2018 Mar 13;8:4470. doi: 10.1038/s41598-018-22564-7 (PMC5849604; doi:10.1038/s41598-018-22564-7)

Tissue Phenomics for prognostic biomarker discovery in low- and intermediate-risk prostate cancer

N.Harder, M. Athelogou, H. Hessel, N. Brieu, M. Yigitsoy, J. Zimmermann, M. Baatz, A. Buchner, C. G. Stief, T. Kirchner, G. Binnig, G. Schmidt, R. Huss

-- Supplemental Material --

Supplemental Figures

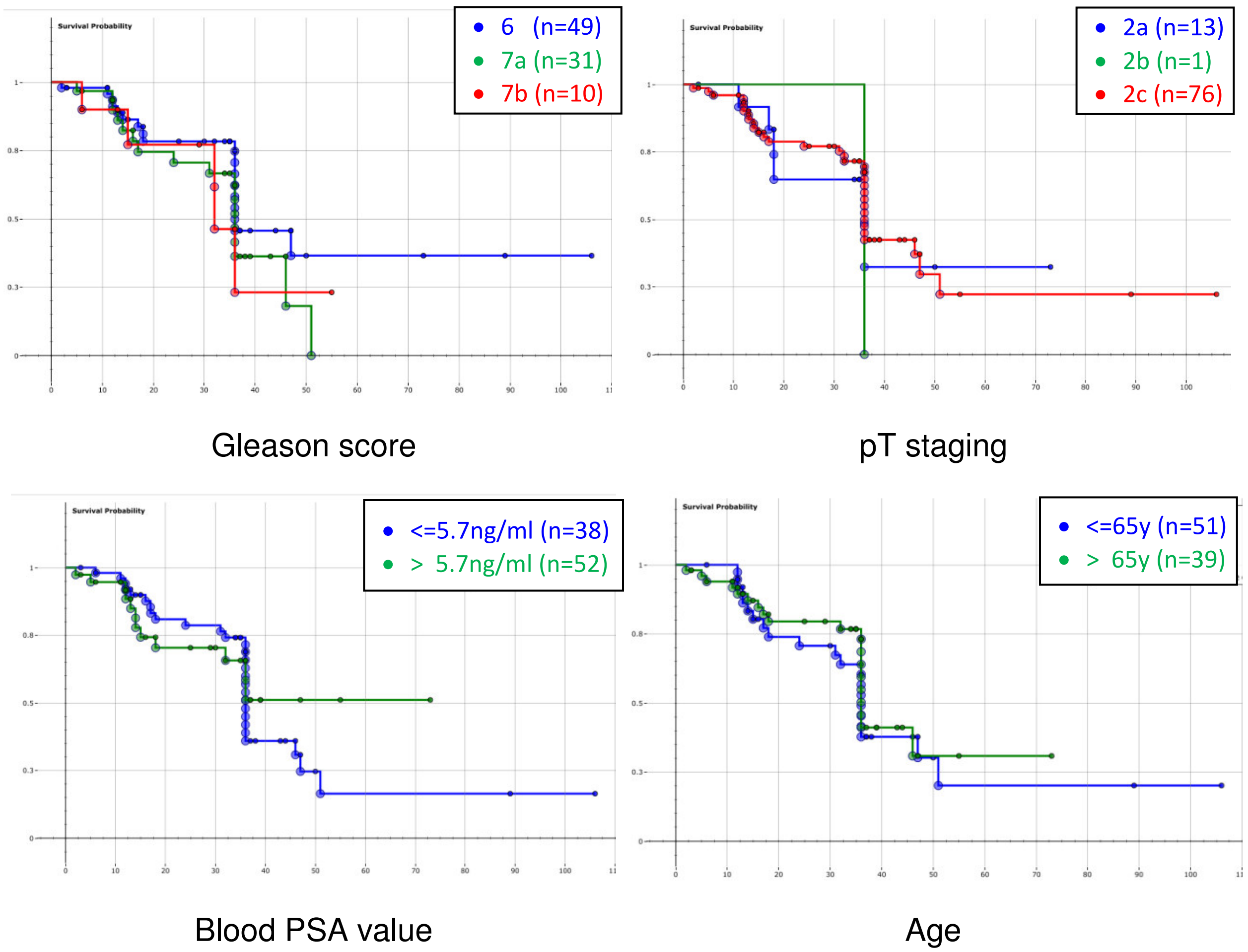

Figure S1: Stratification based on standard clinical data with number of samples per group n in braces. For the blood PSA value and age the cut-off has been optimized to maximize the log-rank test p-value.

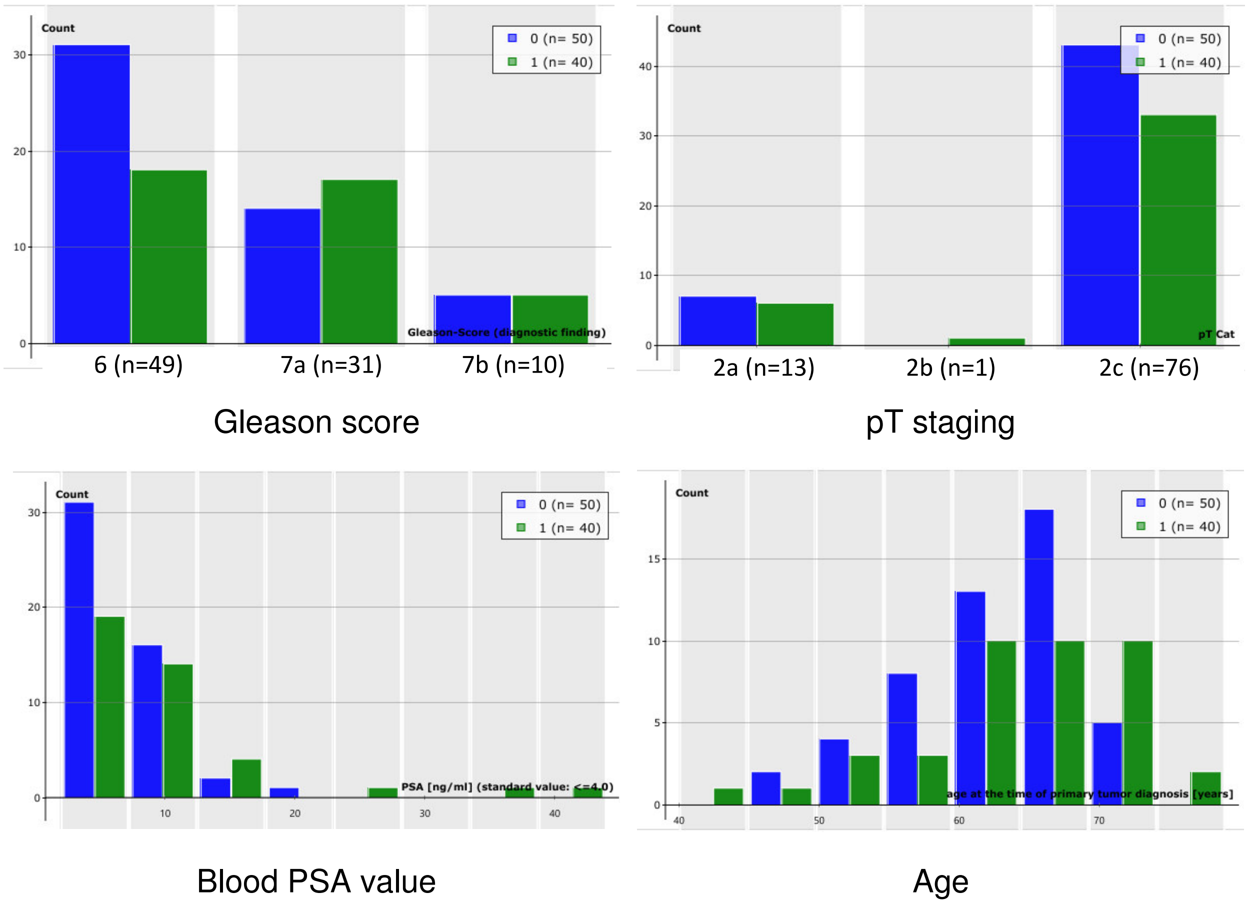

Figure S2: Histograms of standard clinical data over all patients where 0 (blue) refers to the patients without tumor progression and 1 (green) to the patients with tumor progression.

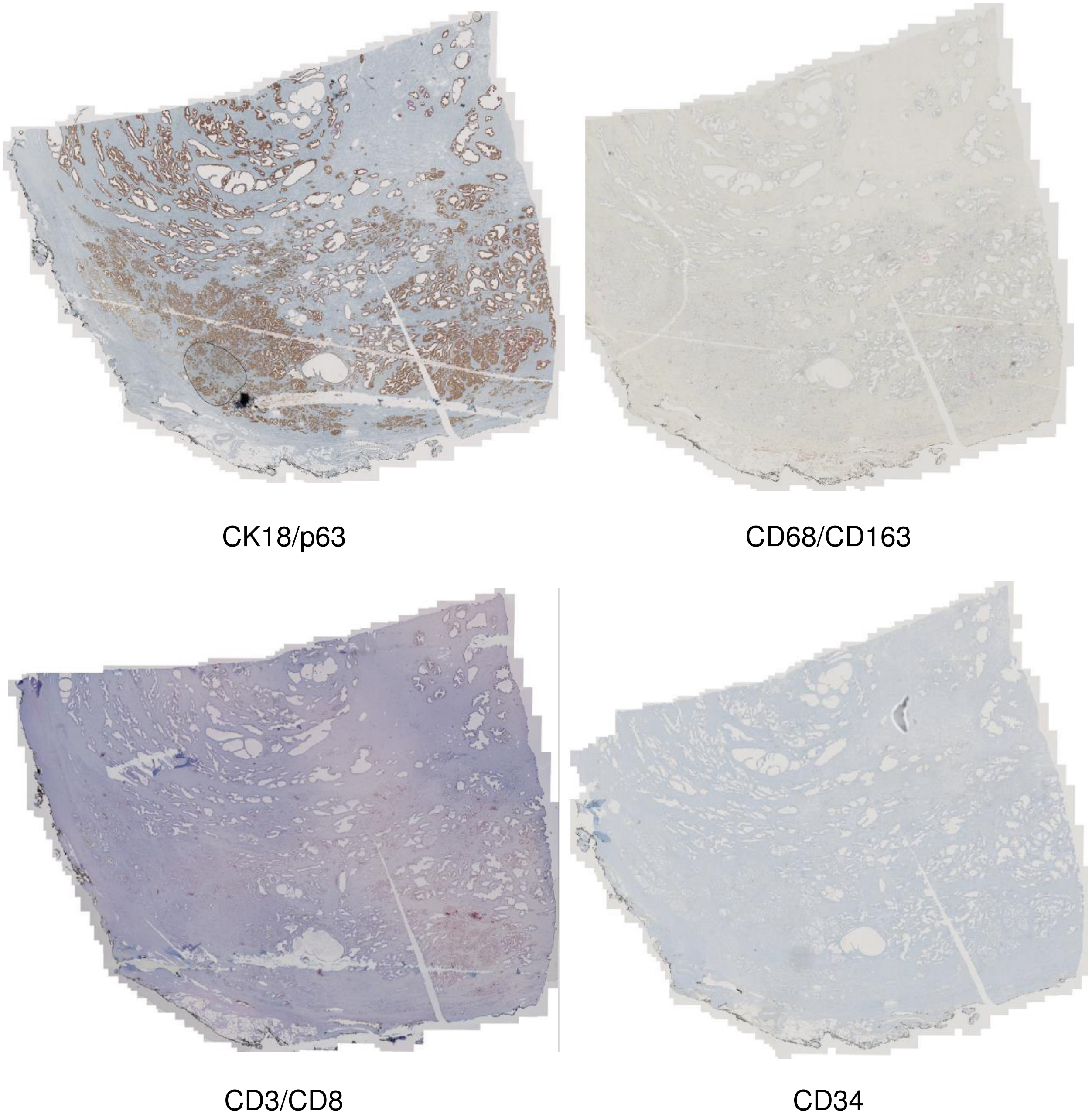

Figure S3: Examples of whole-slide images of consecutive FFPE tissue sections from resected *prostate cancer* tissue.

<table>
<tr><th>Phene</th><th>Kaplan Meier plot</th><th>Accuracy
p-value</th><th>Confusion matrix</th></tr>
<tr><td>1
RATIO
#CD8_border1
TO
#CD34_border1</td><td>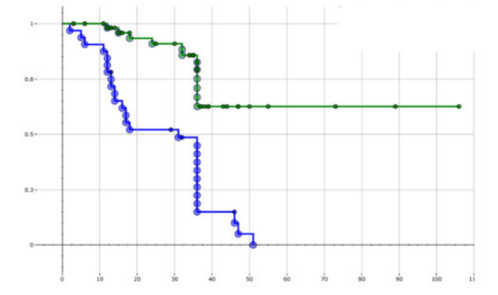</td><td>0.83
3.05e-007</td><td><table><tr><th></th><th>0</th><th>1</th></tr><tr><td>0</td><td>38</td><td>12</td></tr><tr><td>1</td><td>3</td><td>37</td></tr></table></td></tr>
<tr><td>2
DIST CD68 TO
CD34 IN
nonIntact</td><td>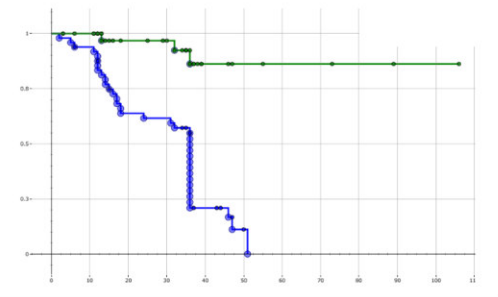</td><td>0.82
2.89e-008</td><td><table><tr><th></th><th>0</th><th>1</th></tr><tr><td>0</td><td>46</td><td>4</td></tr><tr><td>1</td><td>12</td><td>28</td></tr></table></td></tr>
<tr><td>3
CORR Haralick
on intact & non-
intact glands</td><td>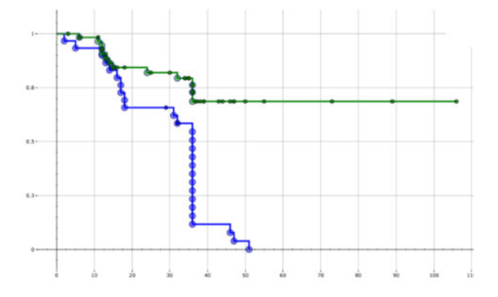</td><td>0.82
4.68e-006</td><td><table><tr><th></th><th>0</th><th>1</th></tr><tr><td>0</td><td>47</td><td>3</td></tr><tr><td>1</td><td>13</td><td>27</td></tr></table></td></tr>
<tr><td>4
RATIO
#CD8_border1
TO
#CD34_border2</td><td>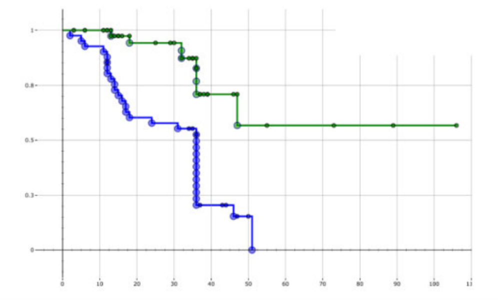</td><td>0.81
2.64e-006</td><td><table><tr><th></th><th>0</th><th>1</th></tr><tr><td>0</td><td>41</td><td>9</td></tr><tr><td>1</td><td>8</td><td>32</td></tr></table></td></tr>
<tr><td>5
RATIO
#CD8_border2
TO
#CD163_border1</td><td>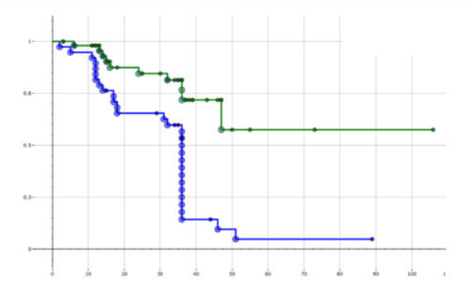</td><td>0.80
2.45e-005</td><td><table><tr><th></th><th>0</th><th>1</th></tr><tr><td>0</td><td>42</td><td>8</td></tr><tr><td>1</td><td>10</td><td>30</td></tr></table></td></tr>
<tr><td>6
DENSITY CD34
IN stroma</td><td>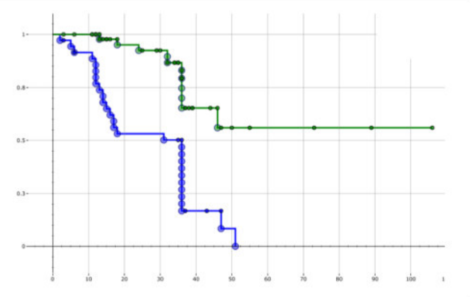</td><td>0.80
4.43e-007</td><td><table><tr><th></th><th>0</th><th>1</th></tr><tr><td>0</td><td>43</td><td>7</td></tr><tr><td>1</td><td>11</td><td>29</td></tr></table></td></tr>
</table>

Figure S4: Result for uni-variate stratification with cross validation using the top-6-ranking significant Phenes in **Table S3**. The Kaplan Meier plots show groups “non-progression” (green) versus “progression” (blue) as predicted, with the events corresponding to true tumor progression. X-axis: disease-free survival time (DFS) in month, y-axis: disease-free survival probability. In the confusion matrices rows provide the true class and columns the predicted class (0: non-progression, 1: progression). (Note that for some patients with DFS times of up to 36 month the exact disease recurrence dates were not available and only the approximated DFS time of 36 month could be used, leading to the event aggregation at 36 month in the plot. For interpretation of the phene names see main manuscript **Table 3**.)

<table>
<tr><th>Method</th><th>Kaplan Meier plot</th><th>Accuracy
p-value</th><th>Confusion matrix</th></tr>
<tr><td>hclust
(ward)</td><td>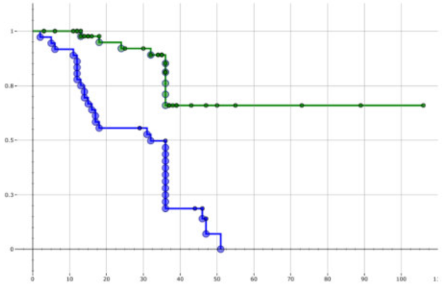</td><td>0.84
1.25e-007</td><td><table><tr><td></td><td>0</td><td>1</td></tr><tr><td>0</td><td>45</td><td>5</td></tr><tr><td>1</td><td>9</td><td>31</td></tr></table></td></tr>
<tr><td>Bayes</td><td>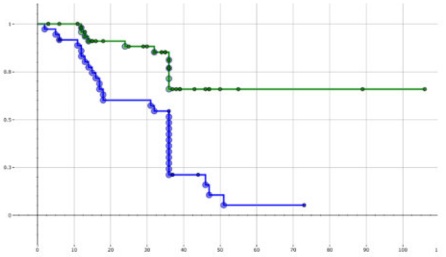</td><td>0.82
7.95e-006</td><td><table><tr><td></td><td>0</td><td>1</td></tr><tr><td>0</td><td>44</td><td>6</td></tr><tr><td>1</td><td>10</td><td>30</td></tr></table></td></tr>
<tr><td>CART</td><td>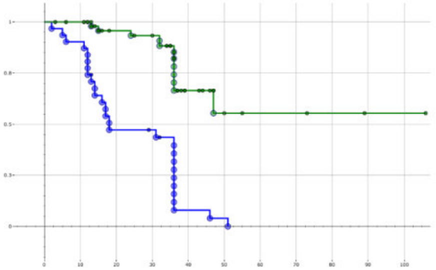</td><td>0.83
2.69e-010</td><td><table><tr><td></td><td>0</td><td>1</td></tr><tr><td>0</td><td>47</td><td>3</td></tr><tr><td>1</td><td>12</td><td>28</td></tr></table></td></tr>
<tr><td>kNN (k=5)</td><td>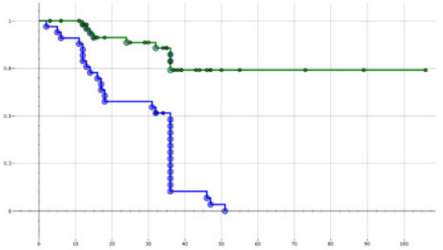</td><td>0.88
3.32e-009</td><td><table><tr><td></td><td>0</td><td>1</td></tr><tr><td>0</td><td>48</td><td>2</td></tr><tr><td>1</td><td>9</td><td>31</td></tr></table></td></tr>
<tr><td>Linear Predictor</td><td>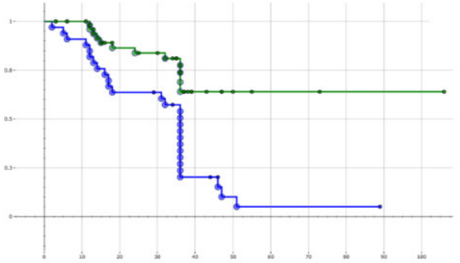</td><td>0.81
2.46e-005</td><td><table><tr><td></td><td>0</td><td>1</td></tr><tr><td>0</td><td>45</td><td>5</td></tr><tr><td>1</td><td>12</td><td>28</td></tr></table></td></tr>
<tr><td>SVM
(lin. kernel)</td><td>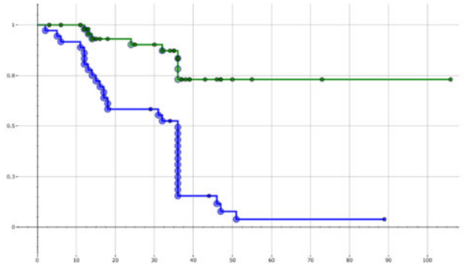</td><td>0.87
5.47e-008</td><td><table><tr><td></td><td>0</td><td>1</td></tr><tr><td>0</td><td>46</td><td>4</td></tr><tr><td>1</td><td>8</td><td>32</td></tr></table></td></tr>
<tr><td>SVM
(rbf kernel)</td><td>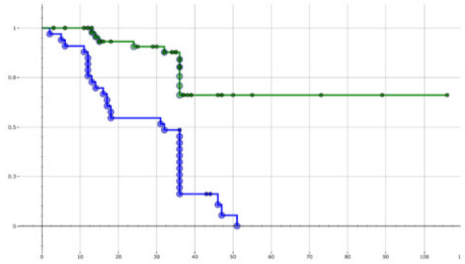</td><td>0.86
3.55e-008</td><td><table><tr><td></td><td>0</td><td>1</td></tr><tr><td>0</td><td>47</td><td>3</td></tr><tr><td>1</td><td>10</td><td>30</td></tr></table></td></tr>
</table>

Figure S5: Performance evaluation of multi-variate stratification using different types of classifiers with the subset of significant Phenes selected using the FWER method of Holm-Bonferroni (**Table S3**). The Kaplan Meier plots show groups "non-progression" (green) versus "progression" (blue) as predicted, with the events corresponding to true tumor progression. X-axis: disease-free survival time (DFS) in month, y-axis: disease-free survival probability. In the confusion matrices rows provide the true class and columns the predicted class (0: non-progression, 1: progression). (Exact disease recurrence dates not available for some patients and therefore the approximated DFS time of 36 month is used.)

Supplemental Tables

	n	Uni-variate			Multi-variate		
		HR	95% CI	p-value	HR	95% CI	p-value
**Gleason Score**							
6	49						
7a	31	1.534	0.788 - 2.987	0.208	1.488	0.714 - 3.103	0.289
7b	10	1.610	0.596 - 4.346	0.348	1.815	0.646 - 5.103	0.258
**pT**							
2a	13						
2b	1	1.582	0.189 - 13.239	0.672	2.648	0.199 - 35.212	0.461
2c	76	0.865	0.361 - 2.077	0.746	0.660	0.256 - 1.705	0.391
**Age**	90	1.020	0.972 - 1.070	0.421	1.024	0.962 - 1.090	0.450
**PSA blood value**	90	1.024	0.984 - 1.066	0.251	1.010	0.964 - 1.058	0.674

Table S1: Result of Cox Regression on clinical features.

IHC	Antibody			Detection systems [1]	Pretreat-ment [1]
	Provider	Dilution	Incubation		
CD68/ CD163	Dako/ Ventana	1:23/ ready to use	40 min/ 16 min	ultraView AP Red/ optiView DAB	32 min, CC1
CD3/ CD8	Zytomed/ Cell Marque	1:150/ 1:50	60 min/ 24 min	ultraView AP Red/ ultraView DAB	60 min, CC1
CD34	Cell Marque	1:200	20 min	ultraView DAB	30 min, CC1
CD18/ p63	Progen / Zytomed	1:200/ 1:200	12 min/ 32 min	ultraView DAB/ ultraView AP Red	60 min, CC1

Table S2: Basic data of IHC protocols

[1] (Ventana Medical Systems, Tucson, Arizona)

	Model: tumor non-progression if<br>Phene	OP	THR	ACC	P-VAL	NUM 5	TP	TN	FP	FN
1	RATIO #**CD8**_*border1* TO #**CD34**_*border1*	>=	0.10	0.83	3.05e-007	81	37	38	12	3
2	DIST **CD68** TO **CD34** IN *nonIntact*	>=	75.70	0.82	2.89e-008	13	28	46	4	12
3	CORR Haralick on intact & non-intact glands	<	0.09	0.82	4.68e-006	0	27	47	3	13
4	RATIO #**CD8**_*border1* TO #**CD34**_*border2*	>=	0.03	0.81	2.64e-006	0	32	41	9	8
5	RATIO #**CD8**_*border2* TO #**CD163**_*border1*	>=	0.65	0.80	2.45e-005	90	30	42	8	10
6	DENSITY **CD34** IN *stroma*	<	0.72	0.80	4.43e-007	0	29	43	7	11
7	DENSITY **CD34** IN *nonIntact*	<	0.85	0.80	2.20e-006	0	28	44	6	12
8	RATIO #**CD8**_*stroma* TO #**CD34**_*stroma*	>=	0.11	0.79	5.19e-006	0	29	42	8	11
9	RATIO #**CD68**_*border2* TO #**CD34**_*innerBorder*	>=	0.07	0.78	1.16e-006	0	28	42	8	12
10	RATIO #**CD3**_*stroma* TO #**CD34**_*border1*	>=	0.45	0.77	0.00038	0	27	42	8	13
11	RATIO #**CD163**_*stroma* TO #**CD34**_*innerBorder*	>=	2.40	0.76	3.59e-006	0	31	37	13	9
12	RATIO #**CD68**_*border2* TO #**CD34**_*border1*	>=	0.04	0.76	1.84e-005	0	25	43	7	15
13	RATIO #**CD8**_*nonIntact* TO #**CD163**_*nonIntact*	>=	0.40	0.74	0.00021	2	28	39	11	12
14	DIST **CD3** TO **CD8** IN *innerBorder*	<	98.69	0.74	0.00047	0	33	34	16	7
15	DIST **CD3** TO **CD8** IN *border2*	<	99.45	0.73	9.71e-005	66	31	35	15	9
16	RATIO #**CD3**_*nonIntact* TO #**CD34**_*nonIntact*	>=	0.18	0.73	0.00034	0	37	29	21	3
17	DIST **CD163** TO **CD8** IN *intact*	<	150.46	0.72	3.15e-005	0	25	40	10	15
18	RATIO #**CD8**_*border2* TO #**CD3**_*nonIntact*	>=	1.57	0.72	0.00012	0	20	45	5	20
19	RATIO #**CD8**_*ws* TO #**CD3**_*ws*	>=	0.73	0.72	7.80e-005	0	22	43	7	18
20	RATIO #**CD163**_*ws* TO #**CD34**_*stroma*	>=	0.93	0.68	0.00022	0	36	25	25	4

Table S3: Ranked list of selected significant Phenes resulting from feature reduction using filtering for accuracy and correlation, followed by FWER multiple-testing correction (Holm 1979). Column **Phene** provides a short definition (for interpretation of the phene names see naming conventions below), which together with the operator **OP** and the threshold **THR** defines the model for classifying patients as tumor non-progression (distances are given in µm). **ACC** provides the cross-validated accuracy, **P-VAL** is the log-rank test p-value of the aggregated prediction, **NUM 5** represents the number of top-5 occurrences over all cross validation runs, and **TP**, **TN**, **FP**, and **FN** gives the true positive, true negative, false positive and false negative predictions, respectively. (For interpretation of the phene names see main manuscript **Table 3**.)

Selected phenes	Uni-variate HR	Uni-variate 95% CI	Uni-variate p-value	Multi-variate HR	Multi-variate 95% CI	Multi-variate p-value
RATIO #**CD8**_*border1* TO #**CD34**_*border1*	8.1e-06	1.3e-08 - 0.0051	**0.00036**	8.9e-04	1.2e-06 - 0.65	**0.037**
DIST **CD68** TO **CD34** IN *nonIntact*	0.99	0.99 - 1	**6.7e-05**	0.998	0.996 - 1.0	0.150
CORR Haralick on intact & non-intact glands	770	61 - 9700	**2.7e-07**	80.1	4.2 - 1531.2	**0.004**
RATIO #**CD8**_*nonIntact* TO #**CD163**_*nonIntact*	0.33	0.13 - 0.82	**0.017**			
DIST **CD3** TO **CD8** IN *border2*	1	1-1	**5.5e-06**			
RATIO #**CD68**_*border2* TO #**CD34**_*border1*	0.0025	2.1e-05 - 0.3	**0.014**			
DIST **CD3** TO **CD8** IN *innerBorder*	1	1 - 1	**7.5e-06**			
RATIO #**CD8**_*ws* TO #**CD3**_*ws*	0.85	0.61 - 1.2	0.340			

A

	n	Multi-variate HR	Multi-variate 95% CI	Multi-variate p-value
RATIO #**CD8**_*border1* TO #**CD34**_*border1*	90	0.001	7.2e-07 - 1.239	0.057
DIST **CD68** TO **CD34** IN *nonIntact*	90	0.997	0.993 - 1.000	**0.034**
CORR Haralick on intact & non-intact glands	90	149.9	5.192 - 4330.060	**0.004**
**Gleason Score**				
6	49			
7a	31	1.110	0.526 - 2.320	0.792
7b	10	2.452	0.832 - 7.229	0.104
**pT**				
2a	13			
2b	1	0.417	0.031 - 5.558	0.508
2c	76	0.533	0.202 - 1.408	0.204
**Age**	90	0.997	0.940 - 1.056	0.905
**PSA blood value**	90	0.981	0.939 - 1.026	0.407

B

Table S4: Result of Cox regression for selected best phenes. (A) Uni-variate Cox regression (left) and multi-variate Cox regression (right, for the top 3 phenes only due to limited number of 40 events). (B) Multi-variate Cox regression of top 3 phenes together with the clinical features. (For interpretation of the phene names see **Table 3**.)

Algorithm Flow Charts

Table of Contents

1) Gland segmentation and classification

2) Cell nucleus segmentation

3) Image co-registration

4) Image feature extraction

5) Feature ranking and phene discovery

Flow Chart Symbols

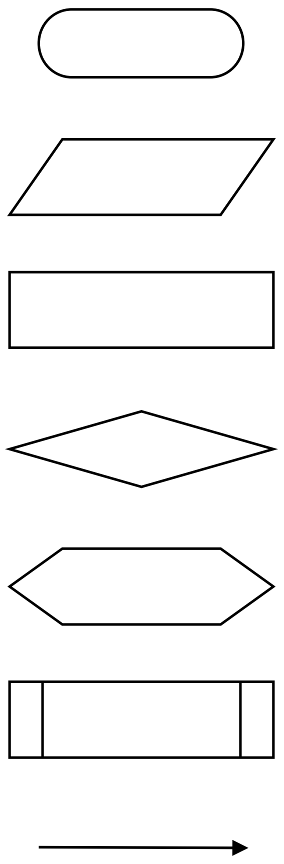

Begin / end

Input / output

Assignment of value / action

Decision

Begin of repetition (loop)

Subroutine

Direction of processing

References

[1] Brieu, N., Pauly, O., Zimmermann, J., Binnig, G., Schmidt, G. "Slide specific models for segmentation of differently stained digital histopathology whole slide images", in *SPIE Med. Imaging* 2016.

[2] Criminisi, A., Shotton, J., Bucciarelli, S., "Decision forests with long-range spatial context for organ localization in CT volumes," in *Medical Image Computing and Computer-Assisted Intervention* (MICCAI) 2009, pp. 69–80.

[3] Forssen, P.-E., "Maximally stable colour regions for recognition and matching," in *Computer Vision and Pattern Recognition* (CVPR) 2007, pp. 1–8.

[4] Sheeba, F., Thamburaj, R., Mammen, J. J., Nagar, A. K., "Splitting of overlapping cells in peripheral blood smear images by concavity analysis," in *Combinatorial Image Analysis*, pp. 238–249 (2014).

[5] Yigitsoy, M. & Schmidt, G. "Hierarchical patch-based co-registration of differently stained histopathology slides", in *SPIE Med. Imaging* 2017.

[6] Zhu, C., Byrd, R. H., Lu, P., Nocedal, J., "Algorithm 778: L-bfgs-b: Fortran subroutines for large-scale bound-constrained optimization," in *ACM Transactions on Mathematical Software (TOMS),* 23(4), pp. 550-560 (1997).

[7] Harder, N. et al. "Co-occurrence features characterizing gland distribution patterns as new prognostic markers in prostate cancer whole-slide images", in *International Symposium on Biomedical Imaging (ISBI)* 2016, pp. 807–810.

[8] Holm, S. "A Simple Sequentially Rejective Multiple Test Procedure", Scand. J. Stat. 6, 65–70 (1979).

[9] Breiman, L., Friedman, J., Stone, C. J. & Olshen, R. A. "Classification and Regression Trees", (Taylor & Francis, 1984).

1) Gland segmentation and classification

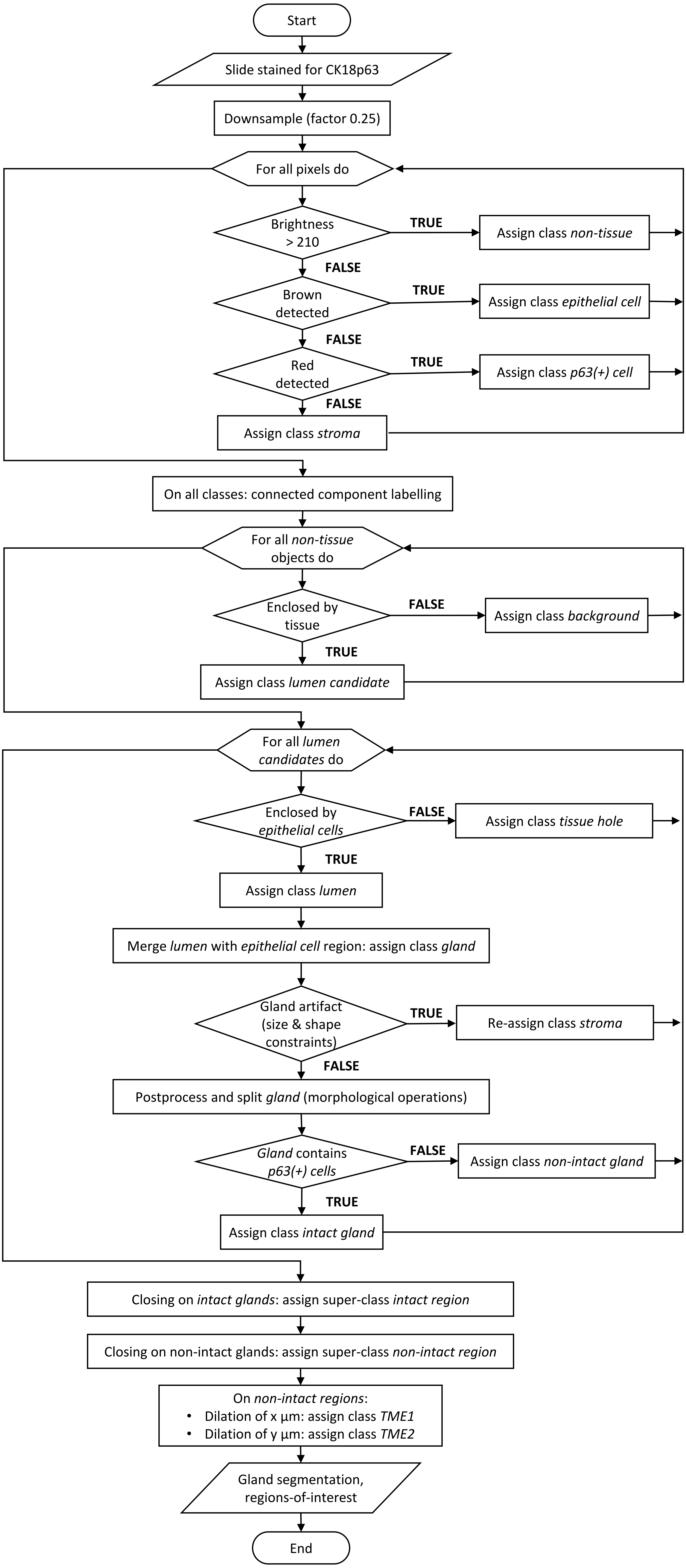

2) Cell nucleus segmentation [1]

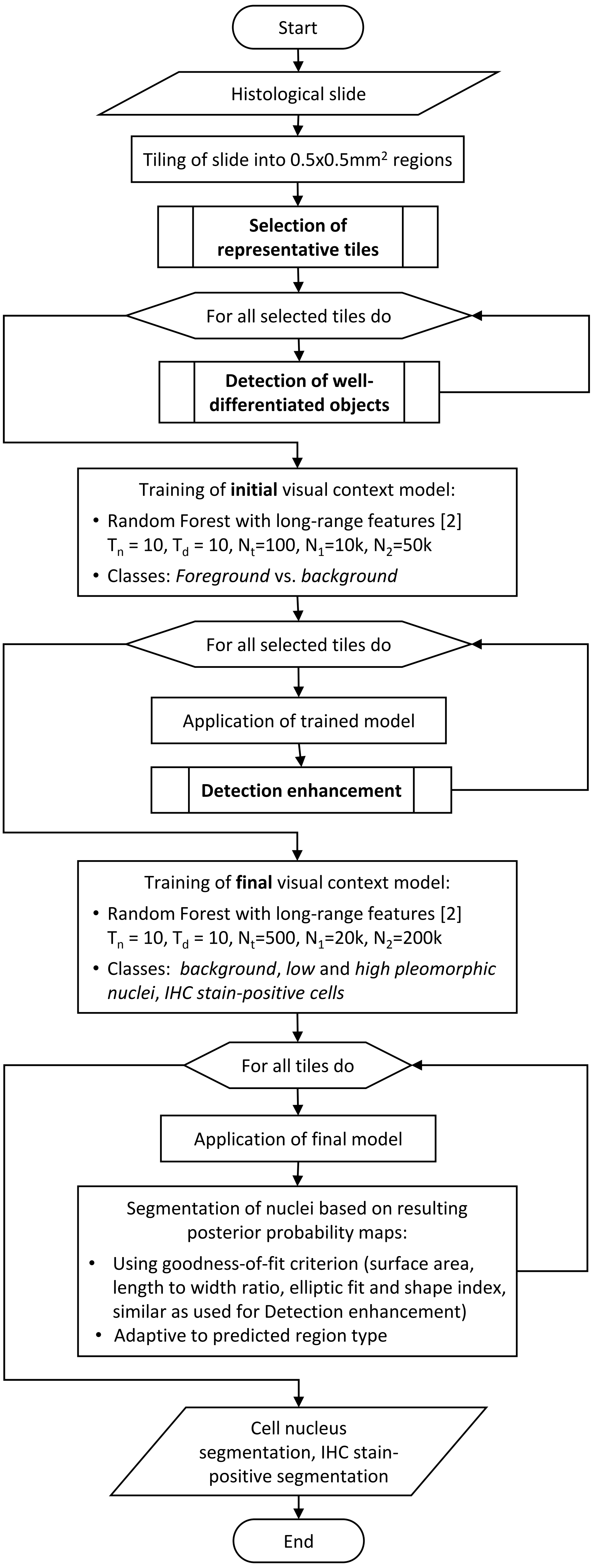

<u>Random forest parameters</u>

T_n : number of trees
T_d : tree depth
N_t : number of tries
N_1: number of random samples used to learn the structure of the random forest
N_2: number of random samples used to refine the thresholds at each node

2.1) Cell nucleus segmentation: Selection of representative tiles

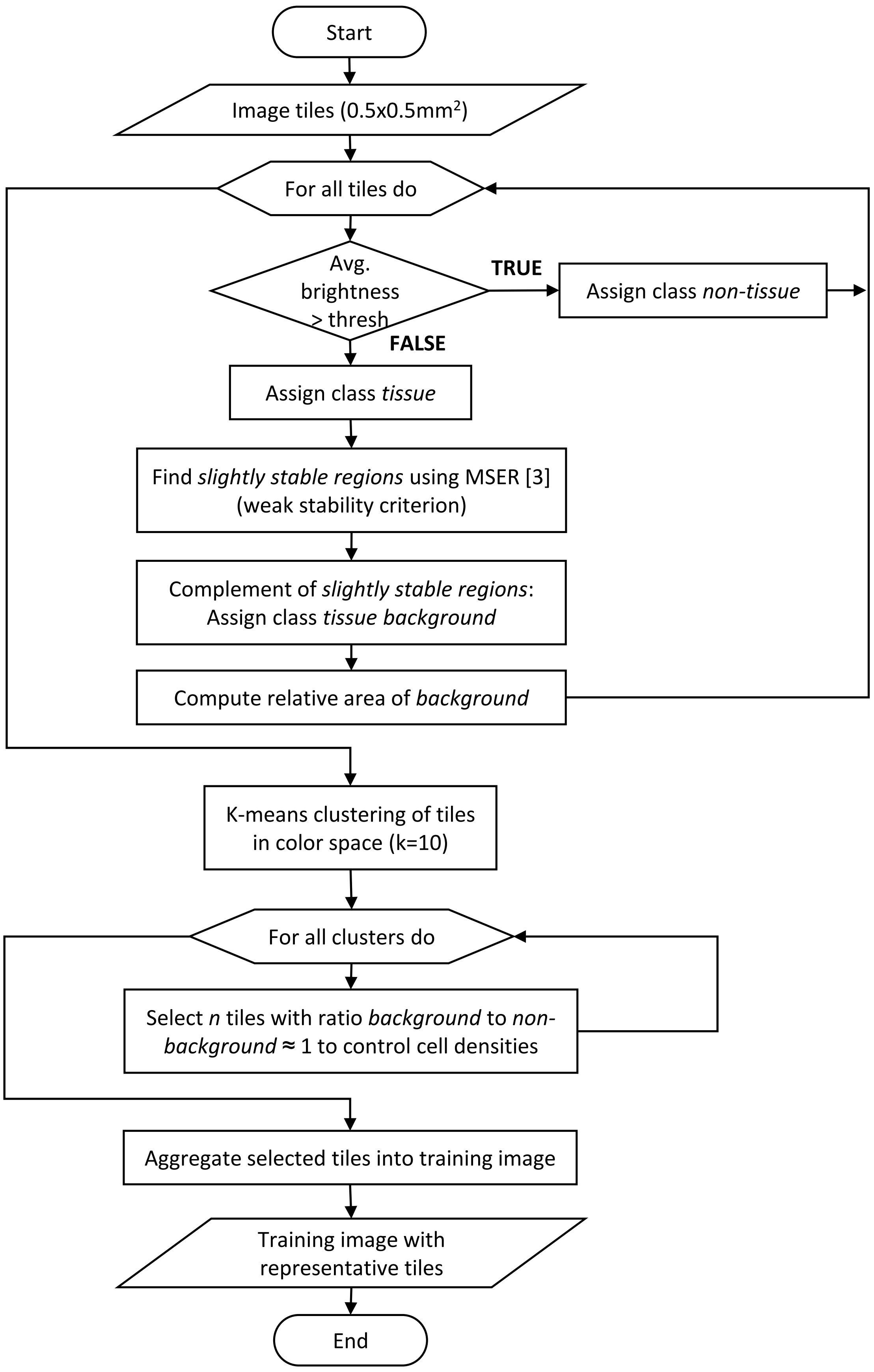

2.2) Cell nucleus segmentation: Detection of well-differentiated objects

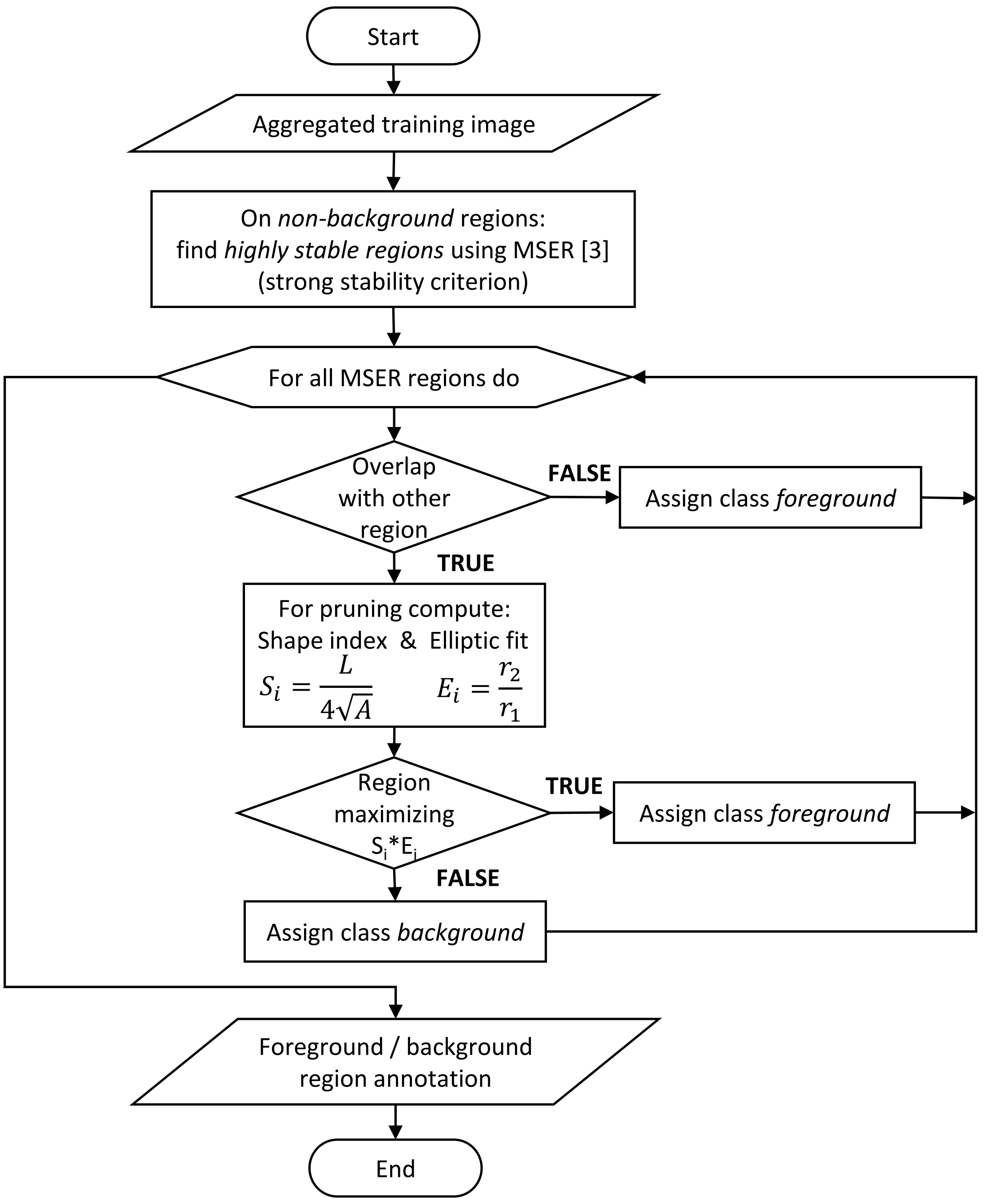

2.3) Cell nucleus segmentation: Detection enhancement

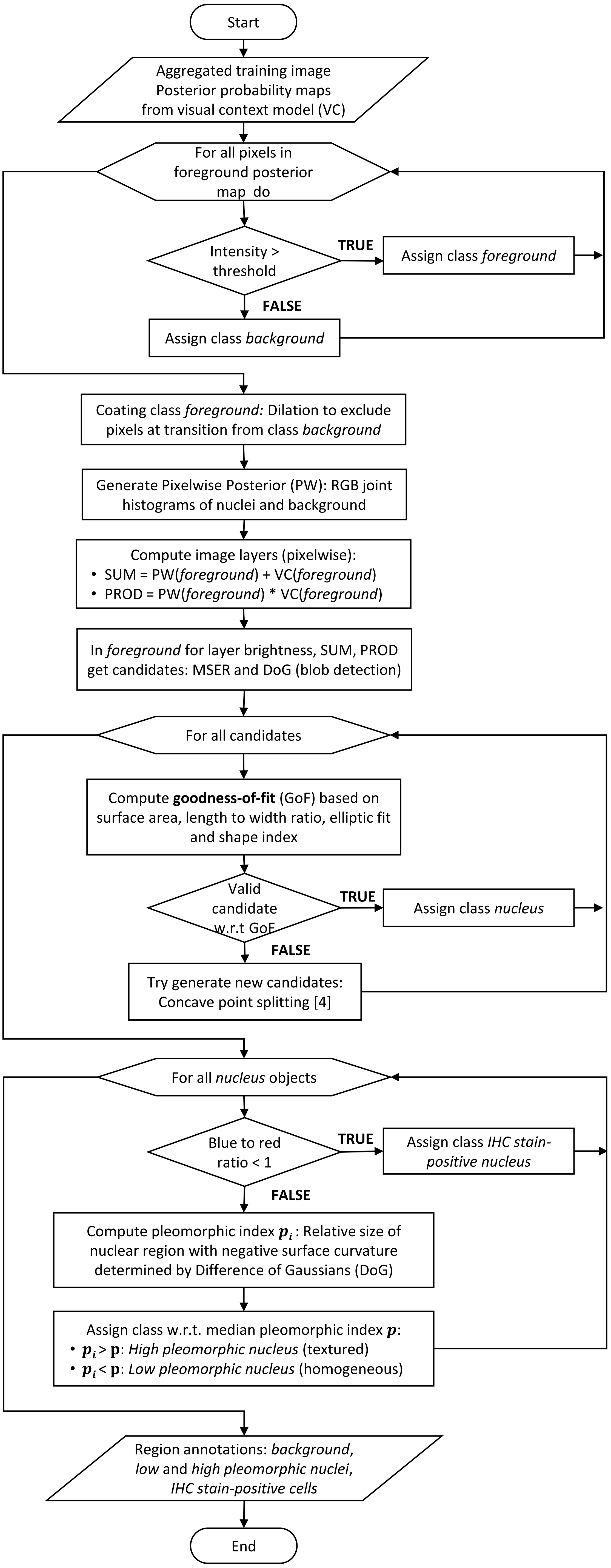

3) Image co-registration [5]

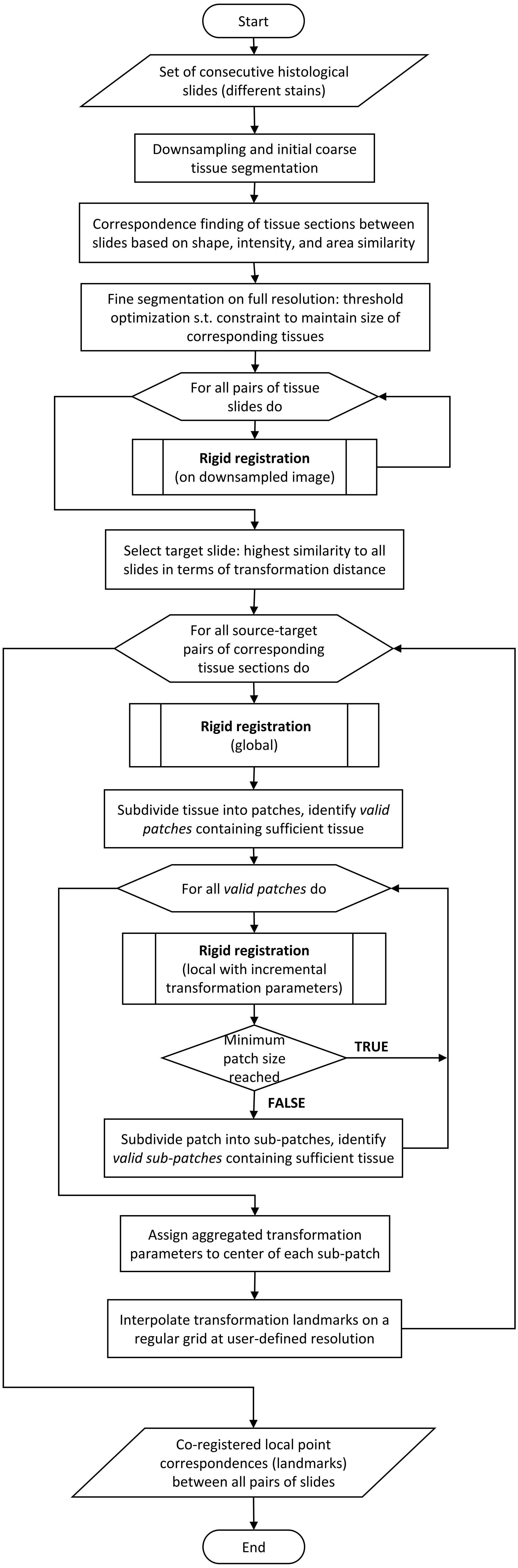

3.1) Image co-registration: Rigid registration

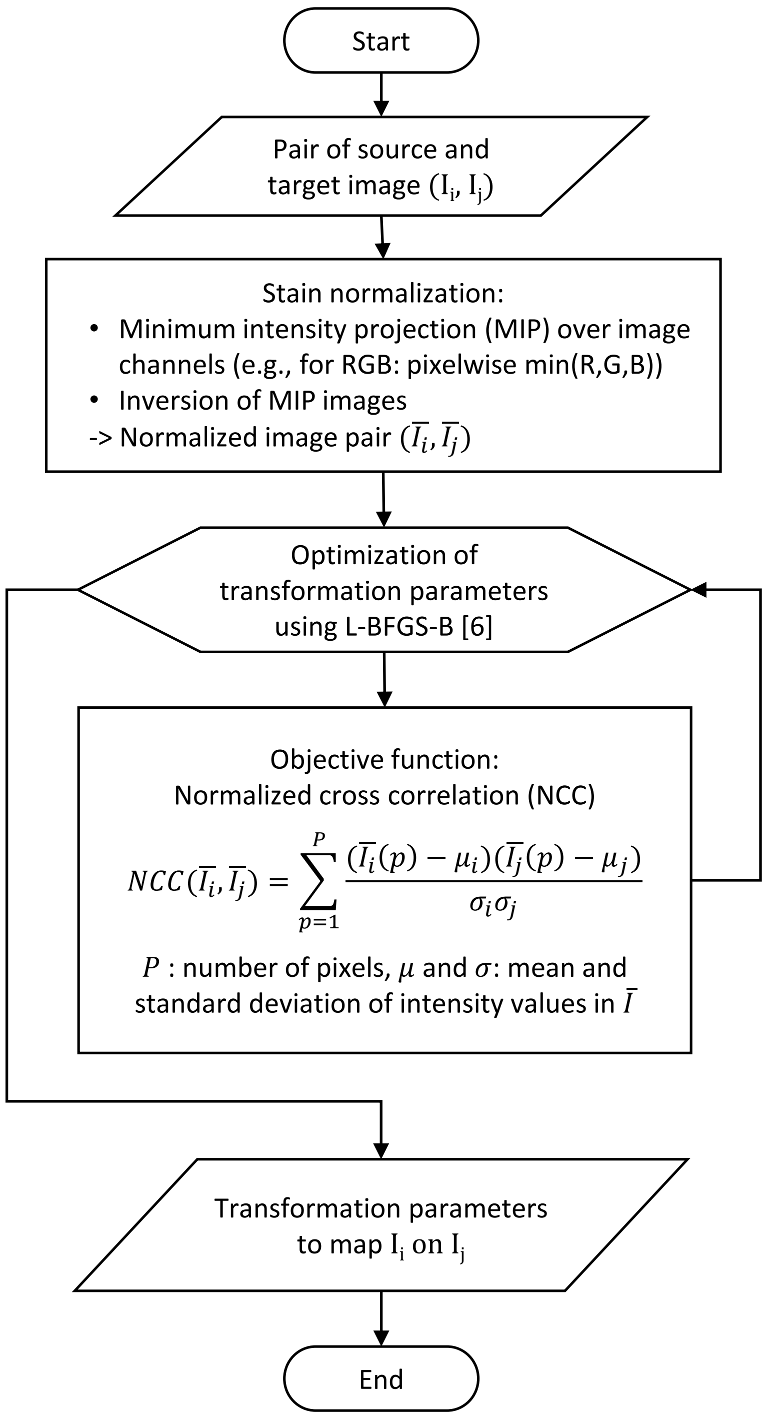

4) Image feature extraction

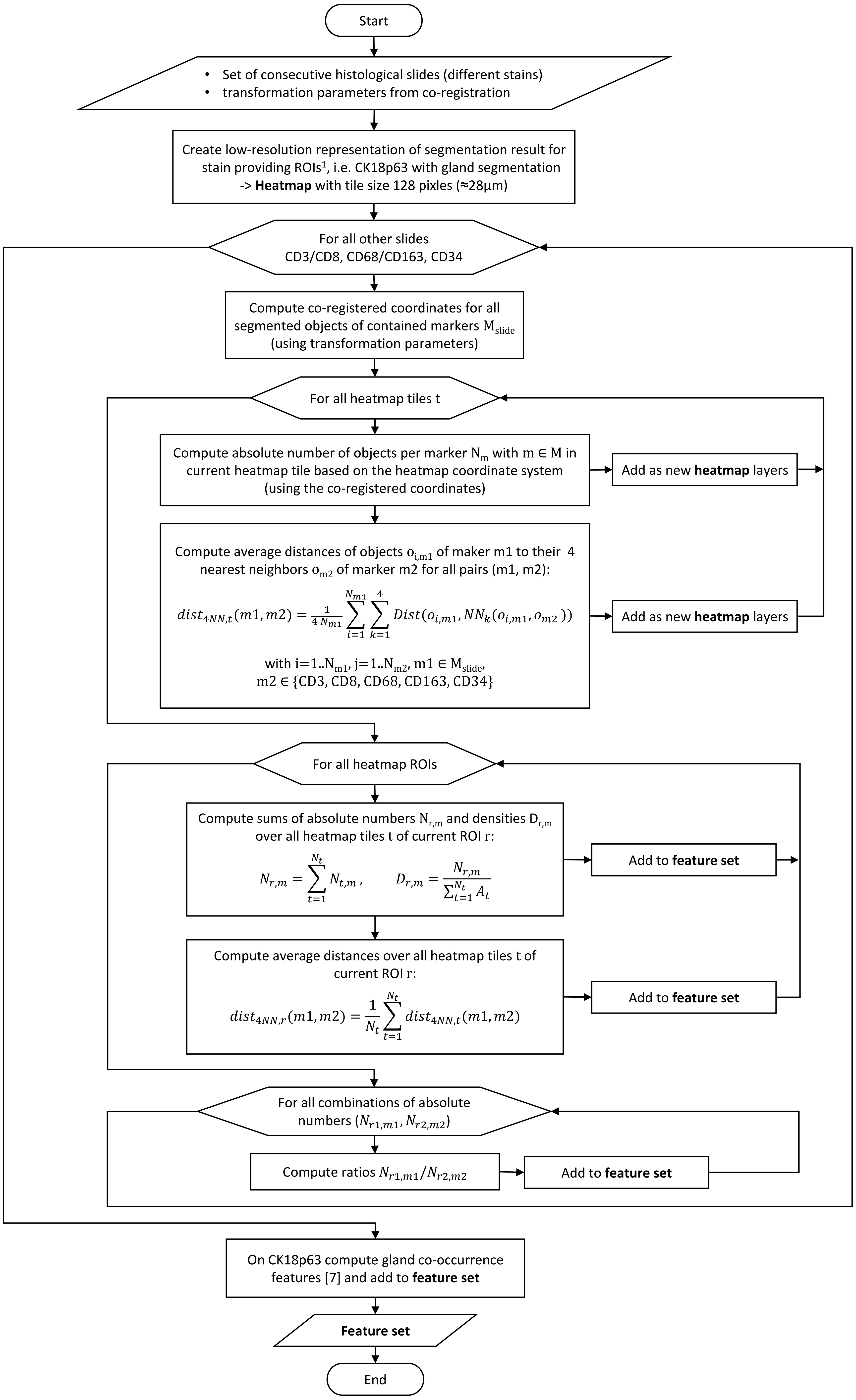

[1]ROIs: Regions-of-interest, i.e., tumor region (non-intact glands), tumor micro environment (TME1, TME2), healthy (intact) glands, stroma

5) Feature ranking and phene discovery

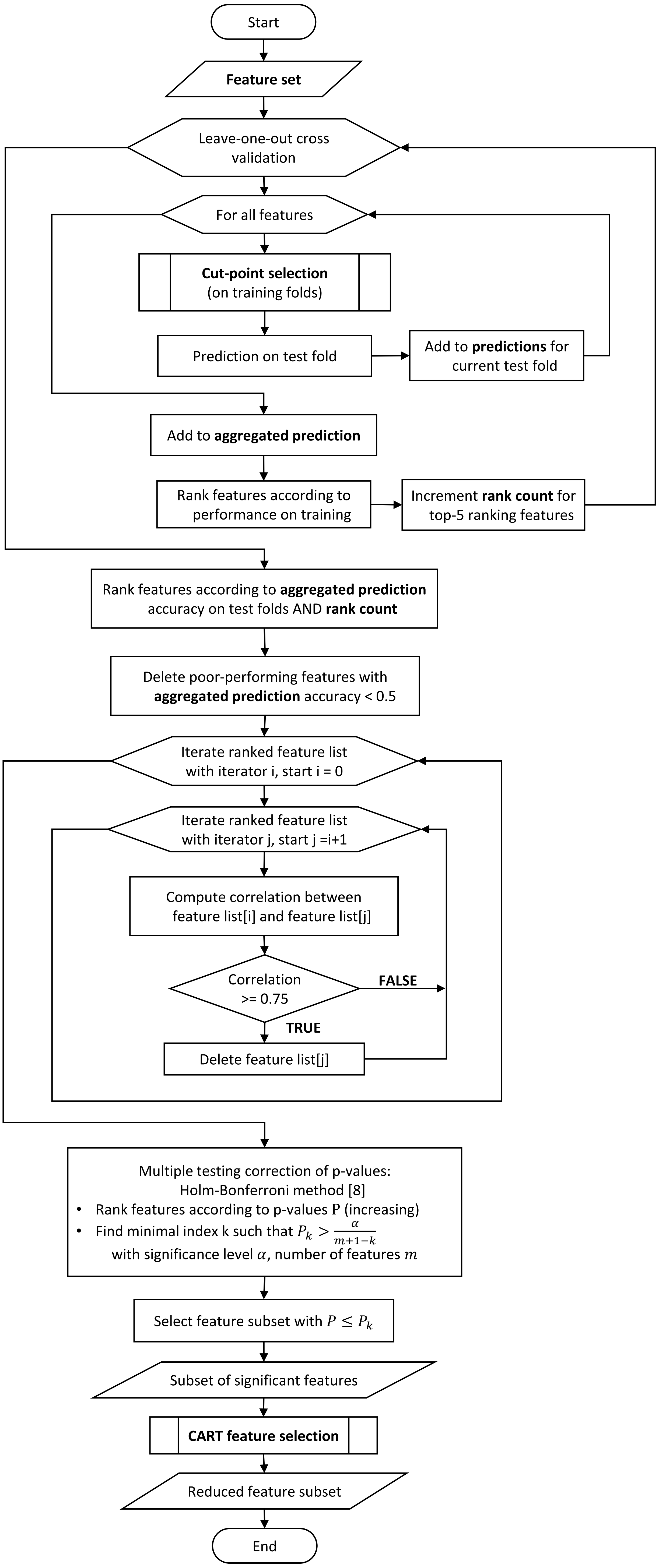

5.1) Feature ranking and phene discovery: Cut-point selection

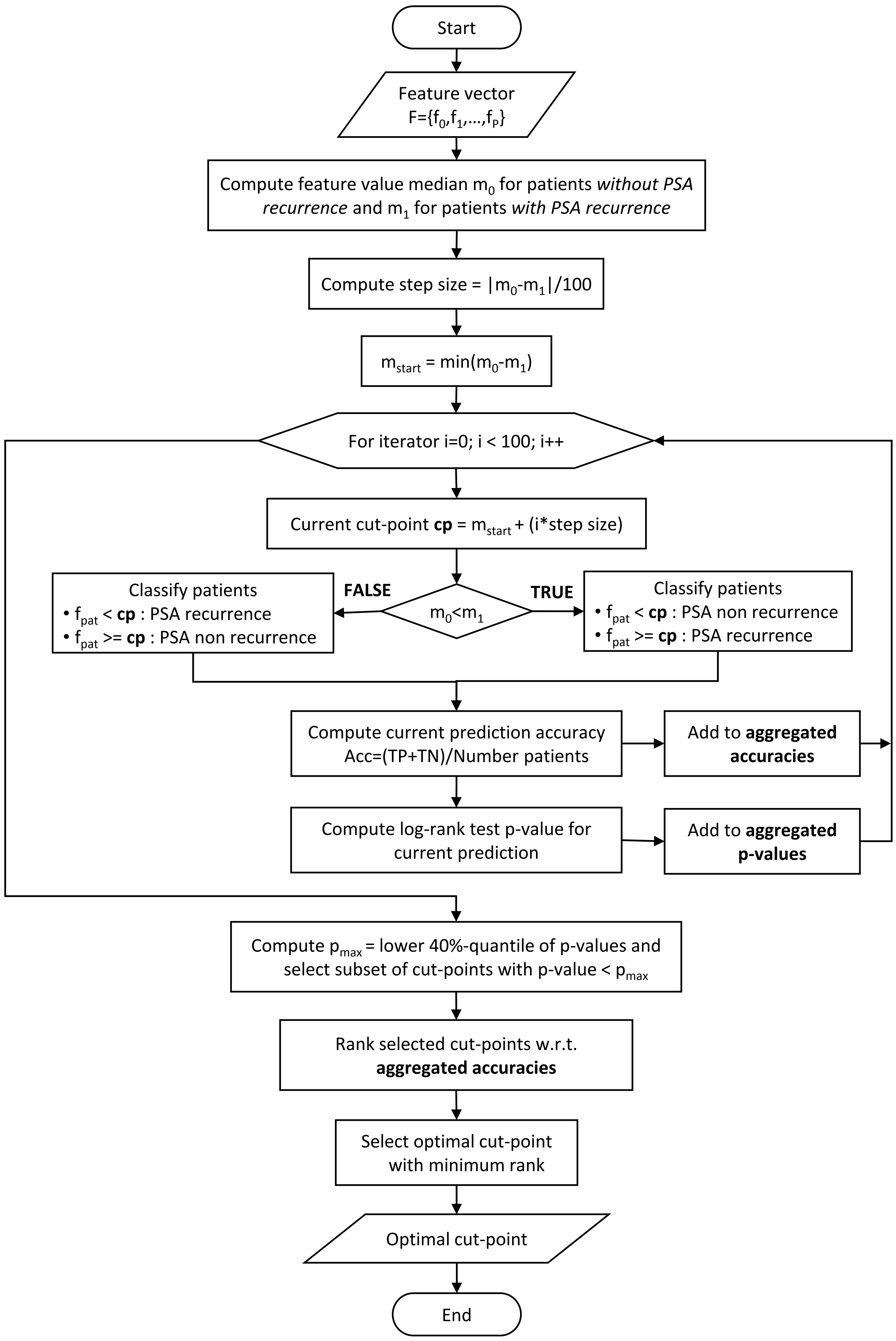

5.2) Feature ranking and phene discovery: CART feature selection

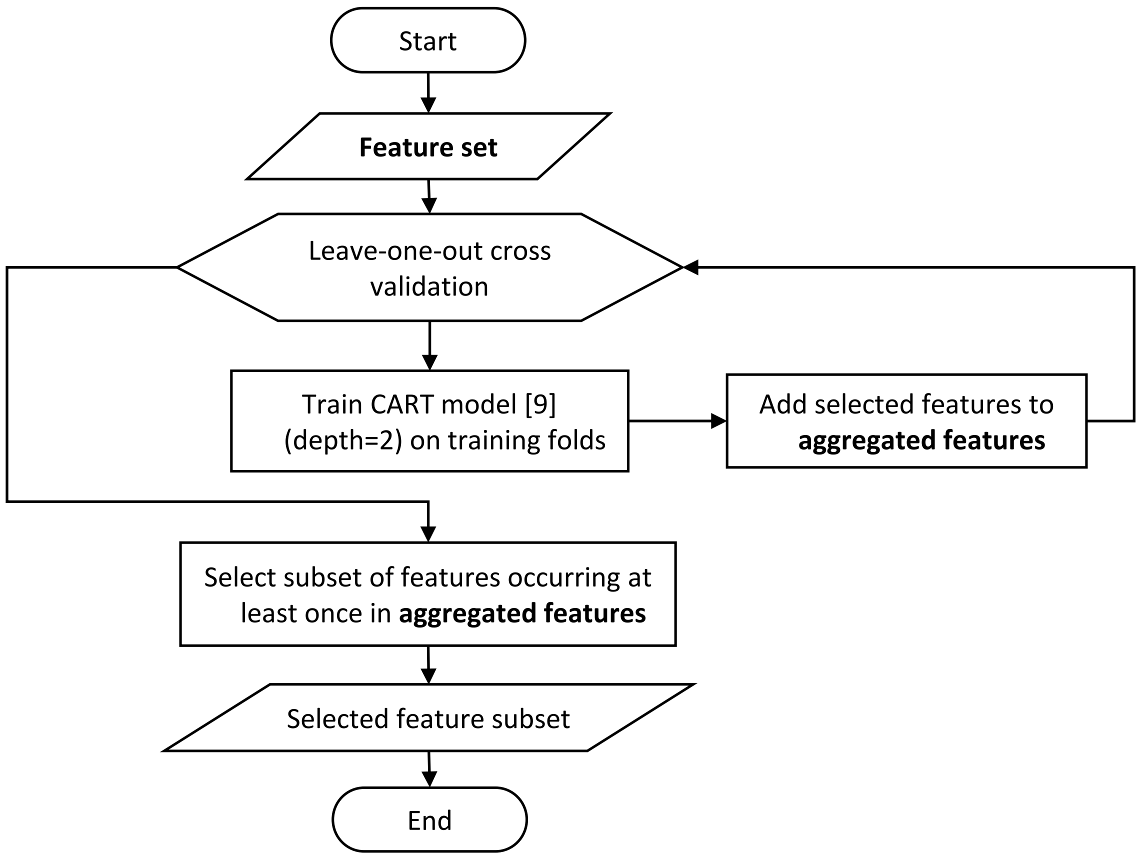

Supplement: Supplementary file 1 — Supplementary Material [file 41598_2018_22564_MOESM1_ESM.pdf]
